# Sialylated and sulfated N-Glycans in MDCK and engineered MDCK cells for influenza virus studies

**DOI:** 10.1038/s41598-022-16605-5

**Published:** 2022-07-26

**Authors:** Lauren Byrd-Leotis, Nan Jia, Yasuyuki Matsumoto, Dongli Lu, Yoshihiro Kawaoka, David A. Steinhauer, Richard D. Cummings

**Affiliations:** 1 Department of Microbiology and Immunology, Emory University School of Medicine, Atlanta, GA USA; 2 Department of Surgery and Harvard Medical School Center for Glycoscience, Beth Israel Deaconess Medical Center, Harvard Medical School, CLS 11087 - 3 Blackfan Circle, Boston, MA 02115 USA; 3 Centers for Excellence in Influenza Research and Surveillance, Emory-UGA CEIRS, Atlanta, GA USA; 4 Department of Pathobiological Sciences, School of Veterinary Medicine, Influenza Research Institute, University of Wisconsin-Madison, Madison, WI USA

**Keywords:** Glycoconjugates, Influenza virus, Glycobiology

## Abstract

The Madin-Darby canine kidney (MDCK) cell line is an in vitro model for influenza A virus (IAV) infection and propagation. MDCK-SIAT1 (SIAT1) and humanized MDCK (hCK) cell lines are engineered MDCK cells that express N-glycans with elevated levels of sialic acid (Sia) in α2,6-linkage (α2,6-Sia) that are recognized by many human IAVs. To characterize the N-glycan structures in these cells and the potential changes compared to the parental MDCK cell line resulting from engineering, we analyzed the N-glycans from these cells at different passages, using both mass spectrometry and specific lectin and antibody binding. We observed significant differences between the three cell lines in overall complex N-glycans and terminal galactose modifications. MDCK cells express core fucosylated, bisected complex-type N-glycans at all passage stages, in addition to expressing α2,6-Sia on short N-glycans and α2,3-Sia on larger N-glycans. By contrast, SIAT1 cells predominantly express α2,6-Sia glycans and greatly reduced level of α2,3-Sia glycans. Additionally, they express bisected, sialylated N-glycans that are scant in MDCK cells. The hCK cells exclusively express α2,6-Sia glycans. Unexpectedly, hCK glycoproteins bound robustly to the plant lectin MAL-1, indicating α2,3-Sia glycans, but such binding was not Sia-dependent and closely mirrored that of an antibody that recognizes glycans with terminal 3-O-sulfate galactose (3-O-SGal). The 3-O-SGal epitope is highly expressed in N-glycans on multiple hCK glycoproteins. These results indicate vastly different N-glycomes between MDCK cells and the engineered clones that could relate to IAV infectivity.

## Introduction

Madin-Darby canine kidney (MDCK) cells are widely used to propagate influenza A virus (IAV)^1,2^ but are unsuitable for amplifying certain strains of IAV. In particular H3N2 viruses, as a consequence of antigenic drift, have altered receptor binding properties that are manifested by their inability to agglutinate either chicken or turkey red blood cells or to grow effectively in MDCK cells^3–6^. Thus, propagation of these viruses in MDCK cells often requires adaptation in the form of a Hemagglutinin (HA) or Neuraminidase (NA) mutation. As such, viruses amplified in MDCK cells are not an accurate facsimile of the original isolate, which has consequences if these cells are used for amplification of candidate vaccine strains. To address these issues, and in general create a more suitable substrate for viruses utilizing a mammalian α2,6-Sia receptor, the MDCK cell line has recently been engineered to express elevated levels of α2,6-Sia. SIAT1 cells overexpress the α2,6-sialyltransferase ST6Gal1^7^, the enzyme responsible for capping complex-type N-glycans with a terminal α2,6-linked Sia. This cell line is more permissive to infection by recent human H3N2 virus strains than WT MDCK cells and do not typically promote sequence changes^5^. Another engineered cell line is the humanized MDCK (hCK) line, which, in addition to overexpressing ST6Gal1, has been engineered to have reduced expression of α2,3-Sia^8^, the canonical avian receptor. The hCK cells are able to propagate strains of H3N2 viruses that have circulated in humans over the past decade and that grow poorly in another ST6Gal1-overexpressing cell line, AX-4.

Because of the reduced ability of IAV H3N2 strains to agglutinate either chicken or turkey erythrocytes^4,9,10^, we sought to identify the glycans present on these blood cells and compare them with the typical N-glycan structures thought to be important for HA binding. We discovered distinct populations of complex N-glycans with a higher concentration of long poly-N-acetyllactosamine-containing structures that are either mono- or di-sialylated in guinea pig red blood cells (RBCs), and conversely, tri- and tetra-antennary branched N-glycans with multiple Sia residues present in chicken RBCs^11^. Guinea pig RBCs can be agglutinated to an extent by these antigenically drifted H3N2 strains. Studies on these strains for interactions with sialylated N-glycans on a microarray revealed that the drifted H3N2 viruses would not bind to either tri- or tetra-antennary glycans with α2,6-Sia as found on chicken RBCs, indicating the importance of the other aspects of glycan structure^11^. Such observations, combined with recent findings suggesting the presence of sulfated glycans on influenza virus derived from multiple infected cell types^12^, and our finding of a preference for phosphorylated glycans in the human lung^13^, indicate that receptors for IAV are more nuanced than predicted.

The MDCK cell lines used to amplify influenza viruses have been tested for Sia expression using the lectins *Sambucus nigra* agglutinin (SNA), capable of binding α2,6-Sia, and *Maackia amurensis* lectin-I (MAL-I), capable of binding α2,3-Sia, but the exact N-glycan structures and composition are not known^7^. Identifying the glycans present in the cell lines will help us to understand more about receptor binding and entry, especially in the context of the H3N2 viruses that have altered receptor recognition characteristics. Here we used mass spectrometry and other approaches to examine the N-glycans isolated from MDCK, SIAT1, and hCK cells at different passages. The results demonstrate significant differences between these cells in the types of sialylated and sulfated N-glycans they generate, which have implications for our understanding of the fundamental mechanisms of IAV infection of cells.

## Results

### MDCK cells express both α2,3- and α2,6-Sia

The N-glycans of total cell extracts were released from glycoproteins by treatment with PNGase F, an enzyme that quantitatively releases N-glycans of all types of structures. Following the permethylation of glycans, MALDI-TOF-MS was used to identify the masses and infer the monosaccharide compositions of glycans^14^. Such information can be used to predict glycan structures, based on prior analyses of N-glycans from mammalian cells and knowledge of the N-glycan biosynthetic pathway^15^. Linkages of Sia were addressed by sensitivity to specific neuraminidases, and predictions of glycan structural features of glycoproteins were tested using specific lectins and antibodies.

**Figure 1:**
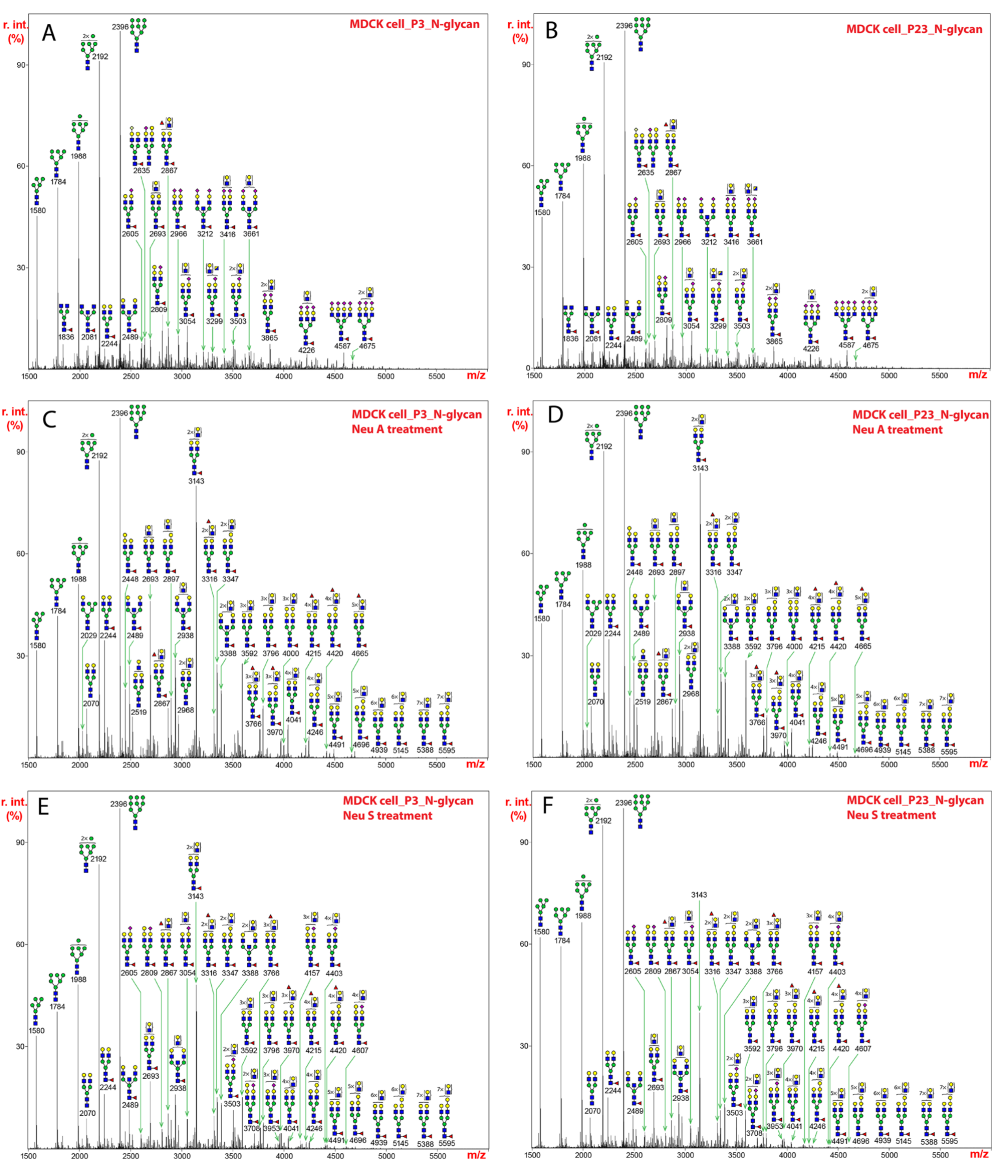
N-glycans released from extracts of MDCK cells by treatment with PNGase F, permethylated, and analyzed by MALDI-TOF MS. The medial molecular range between m/z 1500 and 5500 is shown. Putative structures based on compositions and corresponding to masses detected are represented next to the corresponding mass peaks. (**A**) N-glycans from MDCK cells at passage 3, and (**B**) N-glycans from MDCK cells at passage 23. (**C**) N-glycans in (**A**) from extracts of MDCK cells at passage 3 were treated with neuraminidase A and analyzed. (**D**) N-glycans in (**B**) from extracts of MDCK cells at passage 23 were treated with neuraminidase A and analyzed. (**E**) N-glycans in (A) from extracts of MDCK cells at passage 3 were treated with neuraminidase S and analyzed. (**F**) N-glycans in (B) from extracts of MDCK cells at passage 23 were treated with neuraminidase S and analyzed. The assignments of N-glycan mass and compositions, as well as relative percentage abundance of each glycan species in (**A**), (**C**), and (**E**) are presented in Supplementary Table 3.

The spectrum of such glycans released from MDCK cells at passage 3 revealed that oligomannose-type N-glycans (Man_5-9_GlcNAc_2_) were dominant at the low mass region (m/z 1580, 1784, 1988, 2192, and 2396) (Fig. 1A). The assignments of N-glycan mass and compositions, as well as relative percentage abundance of each glycan species for MDCK cells and other cell lines are presented in Supplementary Table S1. Similar results were seen for N-glycans at passage 23 (Fig. 1B). In the higher mass range, the compositions of a series of peaks were consistent with sialylated and fucosylated complex glycans (e.g., m/z 2605, 2809, 2966, 3054, and 3865) (Fig. 1A,B). Mono-, di-, tri- and tetra-sialylation were all detected and predominantly occurred in the form of Neu5Ac-LacNAc. Most of the complex glycans were mono-fucosylated, which is predicted to represent the common core α1,6-fucosylation. In the high molecular weight region, molecular ions that represent sialylated glycans appear to contain multiple LacNAc repeats (e.g., m/z 3503, 3865, 4226, and 4675). Across the whole mass region, we detected two additional minor structural motifs. A non-human epitope with the composition of Gal-Gal-GlcNAc was observed especially on sialylated glycans (e.g., m/z 2809). This sequence is likely as the Galα1-3Galβ1-4GlcNAc sequence is commonly found at the non-reducing termini in glycans from many mammals and non-human primates^16^. An additional HexNAc, which was proposed to be a bisecting GlcNAc, was found on non-sialylated and sialylated structures (e.g., m/z 2489 and 3212). Additional fucose residues were found in some glycans suggesting an outer modification that might include the H-antigen (e.g. m/z 2867), since these were found on non-sialylated glycans. Altogether these data and predictions are consistent with recent studies of N-glycans on H1N1 derived from MDCK cells^17^, in which oligomannose structures, bisecting GlcNAc and the H-antigen were observed on N-glycans of HA and NA in the virus.

Since sialylated glycoconjugates are a major cellular receptor in the host cell membrane for influenza viruses and the linkage difference is a key factor that determines species barrier, we further characterized the relative levels of α2,3- and α2,6-linked Sia by specific enzymatic treatment. These treatments included replicates of the samples, and we noted the replicates are highly similar. Thus, a single representative image is provided for each MALDI-TOF-MS figure. The relative percentage abundance of each glycan species treated with neuraminidase A or neuraminidase S is presented in Supplementary Table S3. Neuraminidase A, which hydrolyses α2,3-, α2,6- and α2,8-Sia linkages, removed all Sia so that only oligomannose structures and non-sialylated complex N-glycans were observed after the digestion (Fig. 1C,D). All major peaks above m/z 2400 represented complex glycans that were core fucosylated and contained variable lengths of LacNAc (e.g., m/z 2693, 2938, 3143, 3388, 3592, and 4041). Notably, glycans displaying the Gal-Gal-GlcNAc motif were detectable in mid- and high mass regions, which suggested that this non-human epitope is expressed on sialylated glycans with multiple repeats of LacNAc (e.g., m/z 2448, 2897, 3347, 3796).

Neuraminidase S specifically hydrolyses α2,3-Sia linkages. In comparison to the MS profile before treatment, the enzymatic reaction did not significantly alter the N-glycosylation pattern in the mid mass region (Fig. 1E,F). The relative abundances of peaks at m/z 2605, 2809, 3054, 3503, and 3708 remained relatively unchanged. This indicates that sialylated glycans with short LacNAc extensions contain mainly α2,6-Sia and therefore are resistant to neuraminidase S. The elevation of peaks at m/z 2693, 2938, 3143, 3388, 3592, and 4041 represented the formation of desialylation products. In the high mass region, however, Sia was significantly removed by neuraminidase S, indicating that the primary linkages are α2,3-Sia. Most of peaks detected in this region represent non-sialylated species (e.g., m/z 4041, 4215, 4246, 4420, and 4491). The compositions of molecular ions at m/z 4157, 4403, and 4607 represented mono-sialylated glycans with core-fucosylation. These results indicate that the larger N-glycans of MDCK cells contain primarily α2,3-Sia, whereas the smaller N-glycans contain more α2,6-Sia. The relative proportions of sialylated, fucosylated, and/or bisected N-glycans for MDKC cells and the other cell lines are presented in Supplementary Table S2.

### SIAT1 cells predominantly express α2,6-Sia glycans

**Figure 2:**
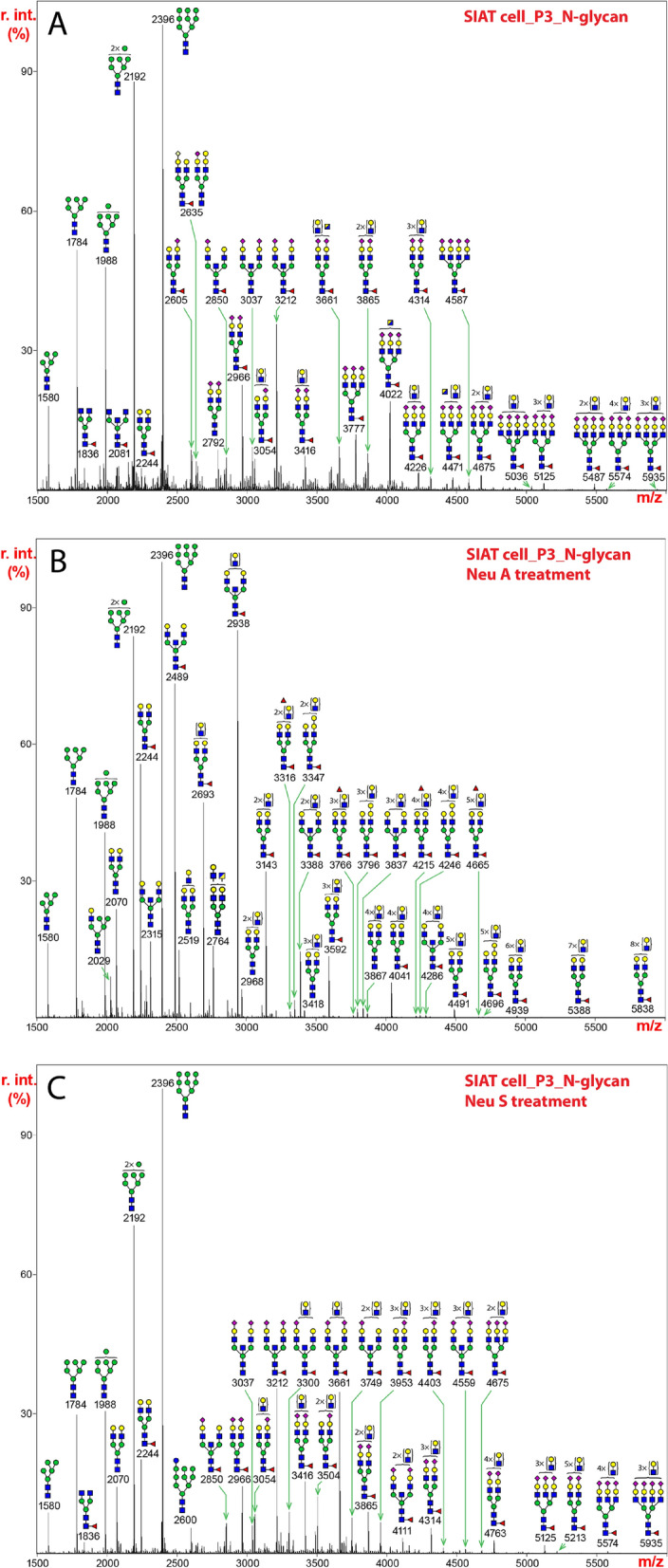
N-glycans released from extracts of SIAT1 cells by treatment with PNGase F, permethylated, and analyzed by MALDI-TOF MS. The medial molecular range between m/z 1500 and 5500 is shown. Putative structures based on compositions and corresponding to masses detected are represented next to the corresponding mass peaks. (**A**) N-glycans from SIAT1 cells at passage 3. (**B**) N-glycans in (**A**) from extracts of SIAT1 cells at passage 3 were treated with neuraminidase A and analyzed. (**C**) N-glycans in (**A**) from extracts of SIAT1 cells at passage 3 were treated with neuraminidase S and analyzed. The assignments of N-glycan mass and compositions, as well as relative percentage abundance of each glycan species in (**A**), (**B**), and (**C**) are presented in Supplementary Table S4.

The N-glycans released from the SIAT1 cell line included a full set of oligomannose type glycans (Man_5-9_GlcNAc_2_) (Fig. 2A), similar to that seen for MDCK cells. Similar spectra were observed for passage 23, indicating as for MDCK cells above, repeated passage (from 3 to 23) did not significantly alter the N-glycan profiles. The abundance of oligomannose type N-glycans appeared significantly higher than complex-type glycans. All major molecular ions detected in the mid and high mass regions represented sialylated species (e.g., m/z 2966, 3212, 3416, 3777, 4022, and 4675). Notably, a prominent glycan at m/z 3212 had the composition of a bisected, sialylated structure. Similarly, the predominant expression of a bisected, sialylated structure at m/z 4022 was also characteristically detected in the high mass region. All of the major complex-type N-glycans were mono-fucosylated, which was proposed to be core-fucosylation, as additional fucose residues were not observed. The levels of fully sialylated, core-fucosylated bi- and tri-antennary glycans at m/z 2966 and 3777 were also higher than that observed in the MDCK cells. (See Supplementary Table S1 for further information).

Neuraminidase A fully released all Sia so that the elevated expression of bisected N-glycan could be more easily visualized (Fig. 2B, Supplementary Table S4). The molecular ion at m/z 2938 represented the most abundant bisected, non-sialylated structure, which was generated following complete removal of Sia from its sialylated form prior to the reaction (Fig. 2A,B, m/z 3661 and 4022). Likewise, the level of the molecular ion at m/z 2489 was notably higher, which arose from de-sialylation of its sialylated form before the enzymatic treatment (Fig. 2A,B, m/z 2850 and 3212). The significant increases in the relative intensities of bisected, de-sialylated species at m/z 2489 and 2938 after neuraminidase A digestion, further confirmed that the elevated expression of bisected, sialylated glycans was characteristic of N-glycans from SIAT1 cells. In addition, the levels of molecular ions at 2244 and 2693, which represented de-sialylated structures, were also higher after the digestion, in comparison to that observed in MDCK cells (Fig. 2B). This feature was consistent with increased abundances of their fully sialylated forms prior to the treatment (Fig. 2A,B, m/z 2966 and 3777).

N-glycans extracted from SIAT1 cells were mostly resistant to neuraminidase S digestion, and almost all major molecular ions observed in the mid and high mass regions represented sialylated glycans (Fig. 2C). The resistance to the enzymatic treatment indicated that SIAT1 cells overwhelmingly express α2,6-Sia. One noticeable feature was that the relative intensity of the molecular ion at m/z 3661, the composition of which was consistent with a di-sialylated, bisected glycan, was elevated after neuraminidase S treatment. This elevation resulted from the loss of one Sia from a tri-sialylated, bisected structure, the molecular ion of which was detected at m/z 4022 (Fig. 2A). Since the molecular ion at m/z 4022 carried three branches with equal length of LacNAc antenna, the linkage of Sia was not equally distributed, which suggested that α2,3- and α2,6-Sia may preferentially occur on specific branches, but this remains to be studied in more detail. Such complexity does not permit us to identify the differences between α2,3- and α2,6-Sia species on specific branches.

### hCK cells predominantly express α2,6-Sia glycans

**Figure 3:**
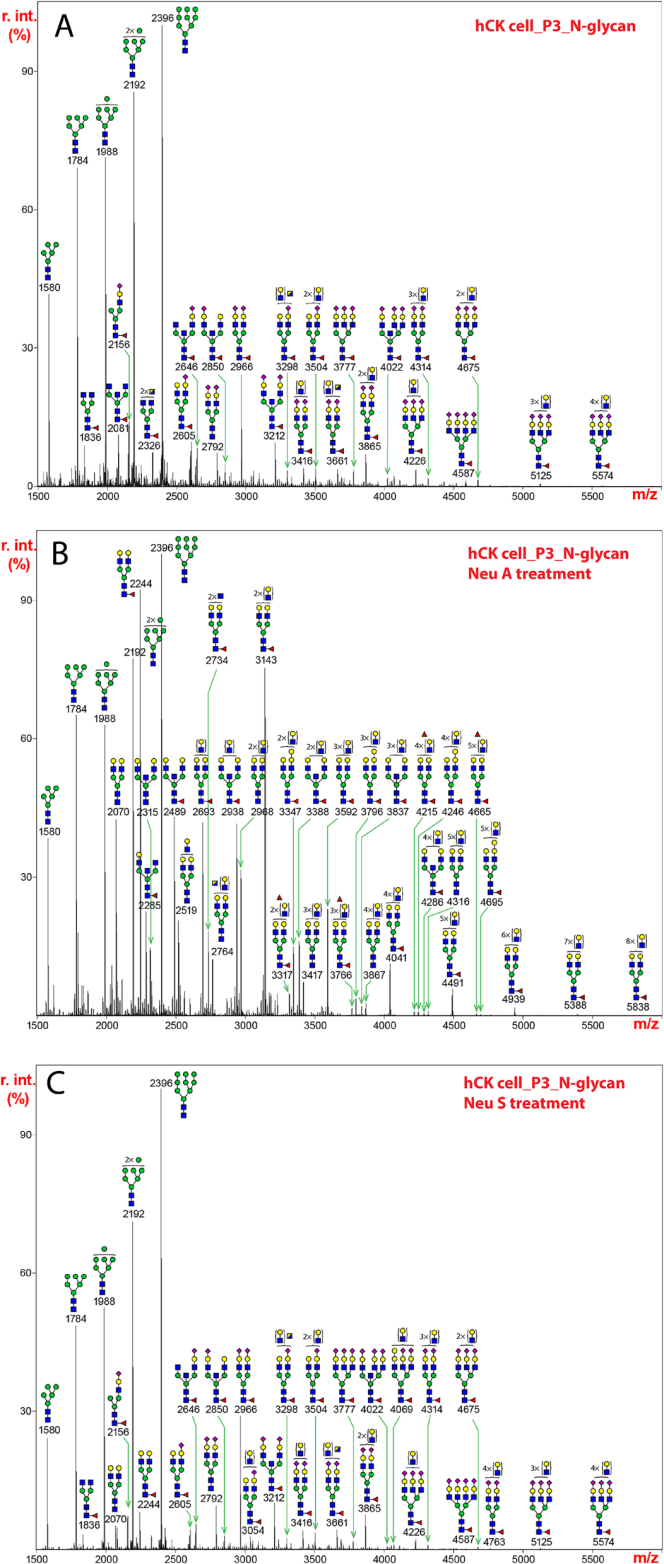
N-glycans released from extracts of hCK cells by treatment with PNGase F, permethylated, and analyzed by MALDI-TOF MS. The medial molecular range between m/z 1500 and 5500 is shown. Putative structures based on compositions and corresponding to masses detected are represented next to the corresponding mass peaks. (**A**) N-glycans from hCK cells at passage 3. (**B**) N-glycans in (**A**) from extracts of hCK cells at passage 3 were treated with neuraminidase A and analyzed. (**C**) N-glycans in (**A**) from extracts of hCK cells at passage 3 were treated with neuraminidase S and analyzed. The assignments of N-glycan mass and compositions, as well as relative percentage abundance of each glycan species in (**A**), (**B**), and (**C**) are presented in Supplementary Table S5.

The MALDI-TOF-MS profile of N-glycans released from the hCK cells was dominated by a full set oligomannose type glycans (Man_5-9_GlcNAc_2_) (Fig. 3A). Again, we observed similar spectra for passage 3 and passage 23, indicating as for MDCK cells and SIAT1 cells above, that the cell lines are stable, and passages did not significantly alter the N-glycan profiles. The composition of the molecular ion at m/z 2966 corresponded to a bi-antennary, di-sialylated, core-fucosylated glycan, which was the most abundant complex-type structure detected. Other major molecular ions observed represented sialylated species with multiple LacNAc units (e.g., m/z 3416, 3504, 3865, 4226, and 4675). Core-fucosylation was a common structural feature, which was observed on most of the major complex-type N-glycans (e.g., m/z 2966, 3212, 3416, 3777, and 4226). Sialylated glycans displaying a bisecting GlcNAc, based on the presence of the additional HexNAc, were also detected (e.g., m/z 3212, 3661, and 4022), but their abundance was not as prominent as that observed in SIAT1 cells (Fig. 2A).

After neuraminidase A treatment, all molecular ions detected corresponded to non-sialylated structures including oligomannose-type glycans and de-sialylated complex glycans (Fig. 3B, Supplementary Table S5). The molecular ion at m/z 2244 represented one of the most abundant reaction products, which was consistent with the prominent expression of its sialylated form prior to the enzymatic digestion (Fig. 3B, m/z 2966).

The MS profile of N-glycans treated with neuraminidase S was similar to that of the untreated glycans (Fig. 3A,C). Almost all sialylated glycans were resistant to the enzymatic digestion, which indicated that the hCK cells exclusively express α2,6-Sia.

### Lectin and mAb blotting for N-glycans and Sia indicate major differences in glycoprotein profiling

To further assess the diversity of glycan modifications and test many of the structural predictions indicated above, as well as the diversity of glycoprotein expression, we performed lectin blotting on cell extracts from passages 3 and 23 with several lectins that recognize N-glycans and their modifications. These include ConA, which binds to hybrid and oligomannose N-glycans, along with biantennary complex-type N-glycans^18–21^; SNA, which can bind glycans with α2,6-Sia^22^; MAL-I, which can bind glycans with either α2,3-Sia^23^ or 3-O-sulfate galactose (3-O-SGal)^24,25^; and PHA-E, which can bind to bisected N-glycans^26^, but not to bisected N-glycans containing α2,6-Sia^27^. Total extracts were either untreated (Mock), treated with PNGase F to remove N-glycans, or treated with neuraminidase A (NeuA) or neuraminidase S (NeuS) to remove Sia, and then analyzed by reducing SDS-PAGE and blotting with the biotinylated lectins above. We also used immunoblotting with a novel monoclonal antibody O6, which binds 3-O-sulfated galactose (3-O-SGal)^28^.

**Figure 4:**
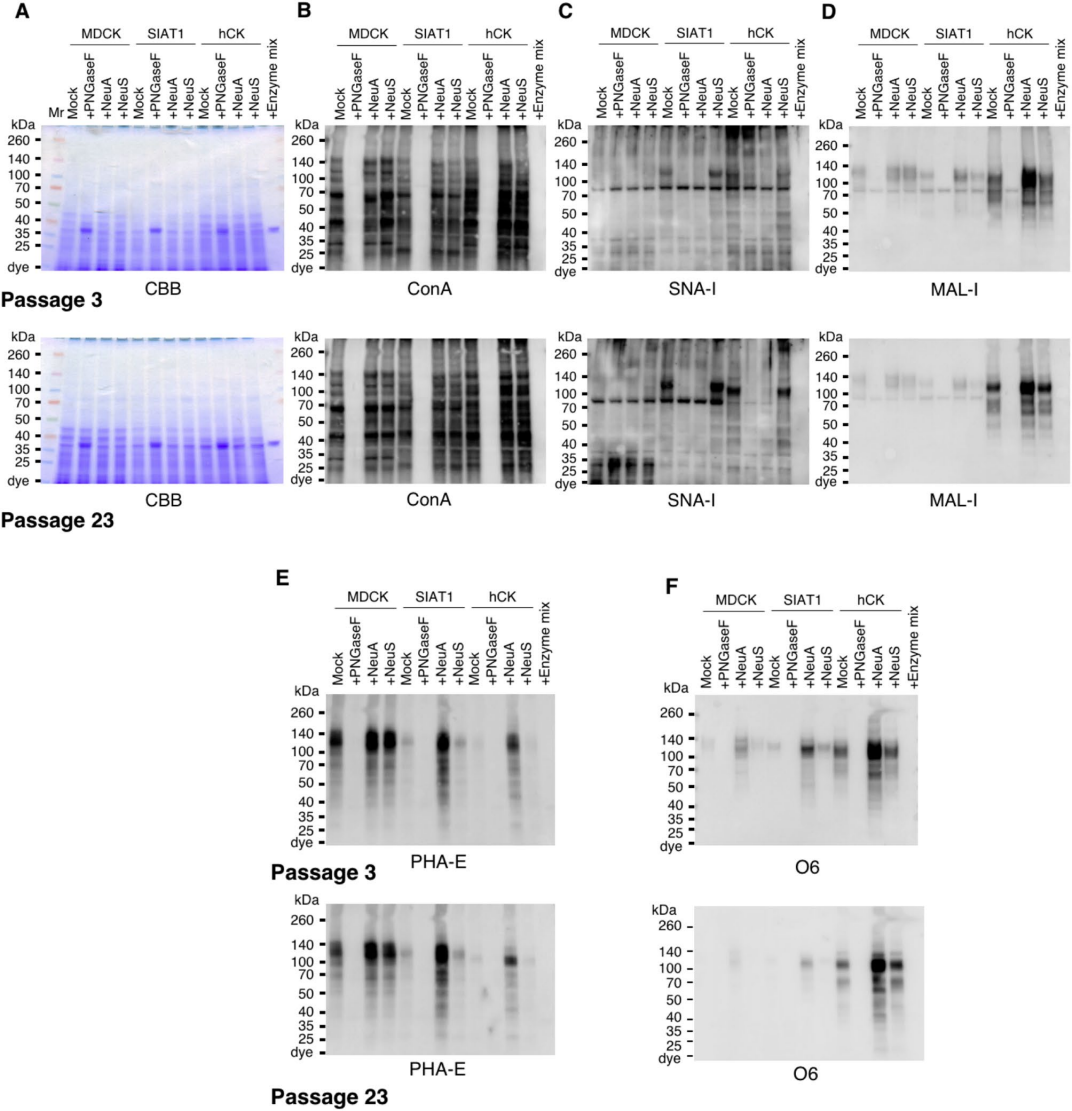
Lectin and Western blot analyses of total glycoproteins in MDCK, SIAT1, and hCK cells. Total cell lysates of MDCK, SIAT1, and hCK cell lines were treated with PNGase F (PNGaseF), Neuraminidase A (NeuA), or Neuraminidase S (NeuS), and separated by SDS-PAGE. Total extracts of cells at passage 3 (top) and passage 23 (bottom) (**A–F**), plus PNGaseF/neuraminidases mixture (Enzyme mix) control were loaded on the gels. (**A**) Gel stained with Coomassie Brilliant Blue solution. (**B**) Transferred total glycoproteins analyzed by lectin blot using ConA; (**C**) SNA; (**D**) MAL-I; and (**E**) PHA-E. (**F**) Total glycoproteins from cells at passage 3, and 23 were Western blotted with the antibody O6.

The results demonstrate that MDCK, SIAT1, and hCK cells express a wide variety of glycoproteins bound by ConA, and binding was largely eliminated by treatment with PNGase F which removes all N-glycans. Similar staining was observed for passage 3 versus 23 (Fig. 4A,B). The patterns of ConA binding to the different cell lines were also comparable, and while some differences were noted between passages, particularly for hCK, overall, the expression patterns were rather similar with only differences in intensities observed.

The binding by SNA and MAL-I was different between the cell lines (Fig. 4C,D). SNA bound to more glycoproteins in hCK cells and with greater intensity compared to MDCK and SIAT1 cells. We note that for SNA, while the patterns of staining were overall similar between passage 3 versus 23, there were some differences which may reflect changes in glycoprotein expression. In addition, unexpectedly, the staining of hCK by MAL-I was much more intense than in MDCK or SIAT1 cells. While the binding of SNA was reduced by neuraminidase treatments, specifically neuraminidase A which cleaves α2,6, the staining by MAL-I was not as significantly affected. This indicates that in SIAT1 and hCK cells, MAL-I is not binding to sialylated glycans. Note that there is a non-specific band in the blots due to the HRP-streptavidin reagent.

Interestingly, for mock-treated material, PHA-E bound to glycoproteins from MDCK cells, but much more weakly to SIAT1 and hCK cells, consistent with the MS results on N-glycans indicating the presence of bisecting N-glycans (Fig. 4E). For MDCK cells, binding of PHA-E was not affected by neuraminidase treatment. However, for SIAT1 and hCK cells, neuraminidase treatments, especially NeuA, significantly exposed more binding sites for PHA-E, suggesting that α2,6-Sia^27^ was blocking binding of PHA-E to N-glycans in those cells. Thus, sialylated, bisecting N-glycans are common in SIAT1 and hCK cells, but these do not bind PHA-E without desialylation.

The result that MAL-I binding to hCK cells was not greatly affected by neuraminidase treatment, suggests that the lectin was binding to glycans not containing α2,3-Sia but instead expressing the 3-O-SGal epitope^24,25^. Indeed, this was directly demonstrated in hCK cells by their strong binding to O6, a monoclonal antibody that specifically binds the 3-O-SGal epitope^28^ (Fig. 4F). Binding was largely eliminated by PNGase F, indicating that the 3-O-SGal epitope is expressed on N-glycans. Importantly, MAL-I can bind glycans with 3-O-sulfated galactose (3-O-SGal)^24,25^, which would be exposed by desialylation. Thus, as might be expected from this prediction, staining by MAL-I and O6 of the hCK cells exhibited many similar features. By contrast, mock-treated MDCK cells bound little O6, but binding was enhanced by treatment with NeuA, which removed all Sia and can expose sulfated epitopes. This finding is similar to what we reported previously for some types of N-glycans, in which they are double modified by both α2,6-Sia and 3-O-SGal, and removal of the α2,6-Sia then exposes the cryptic 3-O-SGal epitope for binding by O6^28^. The staining of MAL-I was not notably different in mock- and NeuS-treated, indicating the presence of O-sulfated glycoproteins, and staining was similar to that seen by O6.

## Discussion

In this study we focused on analyzing N-glycan structures in MDCK cells and two other cell lines derived from them, SIAT1 and hCK, that are engineered to enhance expression of α2,6 sialylated N-glycans. These cells were analyzed as they are important resources for the propagation of many different strains of influenza virus, and analyses of glycans from these cell lines has not been previously documented. Because of studies indicating that human IAV demonstrate a preference for N-glycans with α2,6-Sia, the SIAT1 cells and hCK cells were engineered to express high levels of such α2,6-Sia glycans, through enhanced expression of ST6Gal1, and reduced expression of α2,3-Sia^7,8^. Prior studies on the glycosylation of proteins in these cells were primarily conducted by flow cytometry and reagents to recognize Sia, which indicated a higher level of expression of α2,6-Sia glycans on the cell surface compared to MDCK. For the MDCK cells there was a recent study on the glycosylation of viral glycoproteins HA and NA in H1N1 derived from adherent and suspension-cultured MDCK cells^17^. In that study, the authors reported that the MDCK-derived IAV glycoproteins contain high mannose-type, and fucosylated hybrid- and complex-type N-glycans, including bisected N-glycans. However, because IAV glycoproteins were analyzed, they were not sialylated, likely due to the internal action of the viral NA. Nevertheless, those results are complementary and consistent with our studies on the total cellular N-glycan profiles we observed for MDCK cells.

We used a combination of approaches including MS analysis of PNGase F-released N-glycans, and complementary approaches with lectin/antibody blotting of total glycoproteins with or without exo- or endoglycosidase treatments. The results demonstrate significant differences in N-glycosylation between the cell lines. We also analyzed the cell lines at early and late passages to explore whether passaging affected N-glycan structures or their stable expression. We observed that glycan expression was largely stable for all the cell lines at different passages. This information is important, as there have been studies indicating relatively stability of HA glycosylation during multiple passages of MDCK cells^29^, but the derivative cell lines have not been studied in this regard, and other parameters of cell culture and passage number may affect glycosylation^30^.

While we observed significant differences in N-glycosylation between all three cell lines, one feature that was found in MDCK cell N-glycans and shared by the other cell lines was the prominent expression of high mannose-type N-glycans. The dominance of such glycans at different passages indicate stable expression and unusual regulation of N-glycan processing. Often such glycans are considered intermediates in N-glycan biosynthesis, but the presence of high mannose-type N-glycans on mature viral glycoproteins from MDCK cells are consistent with these being mature structures, and not intermediates^17^.

For complex-type N-glycans, our results demonstrate that MDCK cells exhibit a higher level of α2,3-Sia than α2,6-Sia and a wide variety of complex-type N-glycans that include bisecting structures directly recognized by the lectin PHA-E. By contrast, we found that SIAT1 cells express more α2,6-Sia and are dominated by bisected N-glycan structures. These bisected structures are also present in the hCK cells though in SiaT1 cells they are highly α2,6-sialylated and not recognizable by the lectin PHA-E until they are desialylated. By contrast, the most abundant features in N-glycans of hCK cells were the exclusive presence of α2,6-Sia and the strong expression of 3-O-SGal in N-glycans, as detected by the monoclonal antibody 06 and the binding of the lectin MAL-I to non-sialylated glycans. A dominant N-glycan in the hCK cells has the composition consistent with a biantennary, di-α2,6-sialylated N-glycan possessing a core fucose (m/z 2966). This finding is interesting, as our previous results studying various IAV strains have suggested that such a structure is not a preferred receptor for virus binding^11^.

Together the results of MS analyses and lectin/antibody staining demonstrate significant differences in N-glycosylation between the MDCK, SIAT1, and hCK cell lines. It should be noted that the 3-O-SGal modification was not observed in our MS analyses of N-glycans from these cell lines. In the standard analytical approaches, after glycan permethylation, the glycans are washed with chloroform before further purification with C18 reverse phase chromatography. Thus, sulfated glycans, which are retained in the water fraction, are not analyzed. It is also our experience that sulfated glycans are incompletely methylated and exhibit poor ionization in MS analysis, sulfated moieties are cleaved during the ionization process^31^, and the relative intensities of sulfated glycans, even when detected, cannot be directly compared to non-sulfated glycans. Thus, the structural characterization of sulfated glycans is currently not routinely incorporated into the workflow of glycomics analysis of N-glycans, and their accurate analysis requires more tailored strategies for more rigorous analyses. Our assignment of 3-O-sulfation of galactose is based on specific antibody binding, as this O6 antibody has been extensively defined through crystallography and other methods^28^, and as discussed, assignments of sulfation based on MS analyses is technically challenging as no specific standards exist for such analyses.

The presence of the 3-O-SGal epitope on glycans from MDCK cells and then the enhanced expression of such structures in hCK cells is significant. The hCK cells were engineered to have high expression of the ST6Gal1, which creates the α2,6-Sia, and to have low expression of α2,3-Sia. Our analytical results on glycan structures confirm this expectation. Interestingly, we observed expression of the 3-O-SGal epitope on glycans from MDCK cells, though at a relatively low level, compared to the expression following desialylation in all three cell lines. Unexpectedly, the hCK cells expressed the highest level of the 3-O-SGal epitope after desialylation in comparison to the other lines (Fig. 4F). Such loss of the α2,3-Sia in the hCK cells might explain the higher level expression of the 3-O-SGal epitope, perhaps as a result of loss of enzyme competition for terminal Gal residues of N-glycans, as previously proposed^24^. But there may be additional genetic or epigenetic reasons for the apparent enhanced expression of the 3-O-SGal epitope, and this deserves further analysis. For example, it is possible that expression of the key sulfotransferases that generate the 3-O-SGal may be elevated. The regulation of glycosyl- and sulfotransferase genes is poorly understood. The relevant sulfotransferases for expression of the 3-O-SGal epitope are Gal3ST-2 and Gal3ST-3, both which can sulfate type-2 LacNAc (Galβ1-4GlcNAc) on N-glycans^32^.

The high expression of the 3-O-SGal epitope in SIAT1 and hCK cells is intriguing, because several prior studies have raised the possibility that sulfated glycans may be involved in aspects of viral replication. It has been documented that sulfated glycans are present on influenza virus derived from multiple infected cell types^12^. Sulfation of glycans on IAV has been demonstrated to contribute to significant differences in virus production and replication^33^. The 3-O-SGal modification is also found in the glycolipid sulfatide, whose expression has been shown to enhance efficient replication of IAV, and anti-sulfatide monoclonal antibody was able to protect mice against lethal challenge with IAV^34^. Such results specifically implicate 3-O-SGal in virus replication. A role of sulfated glycans has also been observed in MDCK cells transfected to express glycans with sulfated-6-GlcNAc^35^, which dramatically increased virus production. In addition, there is evidence that N-glycans bearing sulfated-4-GalNAc affect the enzymatic activity of the NA^36^. Interesting, IAV may directly recognize sulfated glycans, as shown for H5N1 interactions with glycans containing α2,3-Sia and 6-O-sulfated GlcNAc residues^37^, and in eggs where H1N1 exhibited high binding to sulfated N-glycans^38^. Direct roles of 3-O-SGal in IAV binding have not yet been explored, but our results suggest this is an avenue for further studies.

Together, these findings suggest that other glycan modifications, beyond Sia alone and including sulfation, may be influential in IAV binding and replication. We recently reported that N-glycans in the human lung are phosphorylated, and such glycans can also be recognized by IAV^13^. Other recent studies indicate that so-called drift strains of IAV demonstrate enhanced preference for such phosphorylated glycans^39^. Thus, the roles of glycan modifications, including sialylation, sulfation, and phosphorylation, may all contribute to efficient propagation of IAV. Our results here suggest that MDCK cells and the engineered cell lines expressing altered sialylated and sulfated glycans, may be useful in exploring these possibilities. In addition, our results indicate that the utility of these engineered MDCK cells for the propagation of these viruses dependent on N-glycosylation pathways is more nuanced than originally expected.

## Methods

### Glycan extraction and purification

Detailed procedures for glycan extraction from biological materials have been described previously^40^. In brief, 1 × 10^8^ cells were sonicated in lysis buffer (25 mM TRIS, 150 mM NaCl, 5 mM EDTA, 1% CHAPS, pH 7.4) to release cellular contents including membrane-bound proteins. Glycoproteins were reduced (inter- and intra-disulfide bonds were reduced) and carboxymethylated by dithiothreitol and iodoacetamide, respectively, before overnight digestion by trypsin. N-linked glycans were released by PNGase F (New England Biolabs, Inc.) and separated from O-glycopeptides by C18 Sep Pak columns (Waters Corp.). All glycans were permethylated and purified by C18 reverse phase chromatography prior to mass spectrometric analysis.

### Neuraminidase treatment

Purified glycans were incubated with neuraminidase A (from *Arthrobacter ureafaciens*, New England Biolabs, Inc.) or neuraminidase S (from *Streptococcus pneumoniae*, New England Biolabs, Inc.) over a period of 24 h at 37 °C prior to permethylation and mass spectrometric analysis.

### MS data acquisition and processing

Permethylated glycans were dissolved in methanol and mixed in equal volume with 2,5-dihydrobenzoic acid (20 mg/ml) before spotting onto a MTP 384 polished steel BC target plate (Bruker Daltonics). Mass spectrometric data was acquired from an UltraFlex II MALDI-TOF Mass Spectrometer (Bruker Corp.) equipped with a Smartbeam II laser. Data acquisition was performed under positive reflectron mode via flexControl (version 3.4, build 135, Bruker Daltonics). Each spectrum shown in the figures represented an accumulation of 20,000 laser shots. Each accumulated spectrum was annotated manually with the aid of flexAnalysis (version 3.4, build 34, Bruker Daltonics) and GlycoWorkBench. The assignment of a glycan composition was based on the ^12^C isotopic composition of a selected peak. All N-glycans detected in the present work were assumed to have a common tri-mannosyl core with the composition of Manα1,6(Manα1,3)Manβ1-4GlcNAcβ1-4GlcNAc. MALDI-TOF-MS was used to identify the masses and infer the monosaccharide compositions of glycans^14^, and with this information the glycan structures can be predicted based on a knowledge of defined mammalian N-glycan structures and biosynthetic pathways^15^. The assignments of N-glycan mass and compositions, as well as relative percentage abundance of each glycan species for MDCK cells and other cell lines are presented in Supplementary Table S1. The relative proportions of sialylated, fucosylated, and/or bisected N-glycans for MDKC cells and the other cell lines are presented in Supplementary Table S2. Relative quantitation was generated using Hex9HexNAc2 as the reference, with relative intensity set to 100%, and the software automatically converted the intensity of all peaks observes in each corresponding MS experiment as a percentile.

This method focuses on evaluating the terminal galactose modifications including sialylation and sulfation of N-glycans from the selected cell lines and their contributions to the infectivity of influenza. Therefore, the SNFG based cartoons for the identified N-glycans with brackets and un-allocated saccharide motifs for LacNAc help to indicate all possible structures (structures that cannot be determined unequivocally) without leading to complications by listing possibly minor and varied structures of each single signal.

The quality of MSMS data for molecular ions above m/z 3000 drops significantly and is unsatisfactory and unreliable for generating defined sequences. In addition, for the majority of molecular ions below m/z 3000, the corresponding glycan structures can be deduced based on the biosynthetic pathways of N-glycans, and therefore MSMS experiments are not performed.

### Cell culture

Madin-Darby Canine kidney (MDCK) cells (ATCC, CCL-34) were maintained in Dulbecco’s Modified Eagle Medium + GlutaMAX (DMEM, Gibco) supplemented with 10% fetal bovine serum (FBS) and 1% Penn/strep (Gibco). MDCK-SIAT1 cells were maintained in DMEM supplemented with 1 mg/ml G418 Geneticin (Gibco), 10% FBS, and 1% Penn/strep. The hCK cell line was maintained in Minimum Essential Media + GlutaMAX (MEM, Gibco) supplemented with 5% newborn calf serum, 1% blasticidin, 1% Penn/strep and 0.02% puromycin. Cells were split at 90% confluency 1:10 into T75cm^2^ flasks and were kept at 37 °C, 5% CO_2_. After desired passage, the culture media was replaced with serum free media in order to reduce the detection of serum glycans, and the flasks were allowed to incubate for 24 h. Cells were released via trypsinization and centrifuged to pellet. The pellet was reconstituted in phosphate buffered saline (PBS) for downstream analysis.

### Lectin and Western blotting

MDCK, SIAT1, and hCK cells (Passage 3 and 23) were lysed with RIPA buffer, and lysates were treated with PNGase F and neuraminidases as described previously^13^. Total protein concentration was determined by Pierce™ BCA assay kit (Thermo Scientific). Samples (30 μg/lane), plus the control of PNGaseF/neuraminidases mixture (Enzyme mix) loaded on SDS-PAGE gels were stained with Colloidal CBB (Bio-Rad), or transferred onto PVDF (EMD Millipore, wet-transfer system, 40 V for 2 h). The membranes were blocked with 5% (w/vol) BSA in TBST for 1 h at room temperature, and incubated with biotinylated lectins ConA (0.1 μg/ml), SNA (2 μg/ml), MAL-I (5 μg/ml), PHA-E (2 μg/ml) (Vector Laboratories), or O6 (1 μg/ml, anti-3-O-SGal antibody; mouse IgG-Fc) in TBST overnight at 4 °C. After washing with TBST, the membranes were incubated with HRP-labeled streptavidin (Vector Laboratories), or goat anti-mouse IgG (H + L) (Jackson ImmunoResearch Laboratories, Inc.) at 1:5,000 dilution for 1 h at room temperature, and the signals were analyzed on an Amersham™ Imager 600 (GE Healthcare Life Sciences) using SuperSignal™ West Pico Chemiluminescent Substrate (Thermo Scientific). See Supplementary Figs. S1 and S2 for original, uncut blots and gels.

## Supplementary Information

Supplementary Table S1.

Supplementary Table S2.

Supplementary Table S3.

Supplementary Table S4.

Supplementary Table S5.

Supplementary Legends.

Supplementary Figures.

## Data Availability

The datasets used and/or analysed during the current study are available from the corresponding author on reasonable request, with no restrictions. The mass spectrometry data has been deposited in an appropriate online database available at: https://glycopost.glycosmos.org/entry/GPST000269.
