# Supplementary material for: Sialylated and sulfated N-Glycans in MDCK and engineered MDCK cells for influenza virus studies
Source: Sci Rep. 2022 Jul 26;12:12757. doi: 10.1038/s41598-022-16605-5 (PMC9325728; doi:10.1038/s41598-022-16605-5)
Supplement: Supplementary file 1 — Supplementary Table S1. [file 41598_2022_16605_MOESM1_ESM.pdf]

Supplementary Table 1

List of assigned peaks of N-glycans by MALDI-TOF-MS analysis in cell lines

Composition	MDCK		SIAT1		hCK		Remarks
	Mass (m/z)	%	Mass (m/z)	%	Mass (m/z)	%	
Hex_5 $HexNAc_2$	1579.8	50.03	1579.9	20.96	1579.9	43.64	
Fuc_1 Hex_3 $HexNAc_3$	1590.8	1.35	1590.9	0.89	1591.0	3.14	
Hex_4 $HexNAc_3$	1620.9	0.84	1620.9	0.25	1621.0	2.55	
Hex_6 $HexNAc_2$	1784.0	57.73	1784.1	55.89	1784.2	64.88	
Fuc_1 Hex_4 $HexNAc_3$	1795.1	1.84	1795.1	0.43	1795.2	3.58	
Fuc_1 Hex_3 $HexNAc_4$	1836.1	8.11	1836.2	5.51	1836.3	12.58	
Hex_4 $HexNAc_4$	1866.1	1.03	1866.2	0.90	1866.3	1.90	
Hex_3 $HexNAc_5$	1907.2	0.93	1907.3	0.95	1907.3	4.31	
Fuc_2 Hex_4 $HexNAc_3$	1968.2	0.55	1968.3	0.57	1968.3	0.38	
$NeuAc_1$ Hex_4 $HexNAc_3$	1982.3	0.83	1982.4	0.97	1982.4	4.24	
Hex_7 $HexNAc_2$	1988.2	65.65	1988.3	52.65	1988.4	73.28	
Fuc_1 Hex_4 $HexNAc_4$	2040.3	1.76	2040.4	1.18	2040.5	2.35	
Hex_5 $HexNAc_4$	2070.3	5.64	2070.4	5.26	2070.5	5.31	
Fuc_1 Hex_3 $HexNAc_5$	2081.3	7.00	2081.4	5.37	2081.5	14.62	
Hex_4 $HexNAc_5$	2111.3	1.43	2111.4	0.83	2111.5	1.71	
$NeuAc_1$ Fuc_1 Hex_4 $HexNAc_3$	2156.4	4.09	2156.5	2.88	2156.5	17.68*	*Sialylated product
$NeuAc_1$ Hex_5 $HexNAc_3$	2186.4	4.88	2186.5	5.33	2186.6	6.42	
Hex_8 $HexNAc_2$	2192.4	93.62	2192.5	88.87	2192.5	88.59	
Fuc_2 Hex_4 $HexNAc_4$	2214.4	1.37	2213.5	1.09	2213.5	0.72	
$NeuAc_1$ Hex_4 $HexNAc_4$	2227.4	0.98	2227.5	1.22	2227.6	3.18	
Fuc_1 Hex_5 $HexNAc_4$	2244.4	5.61	2244.5	4.60	2244.6	5.61	
Hex_6 $HexNAc_4$	2274.4	2.59	2274.5	1.45	2274.6	1.31	
Fuc_1 Hex_4 $HexNAc_5$	2285.4	2.62	2285.5	1.07	2285.6	2.81	
Hex_5 $HexNAc_5$	2315.4	3.77	2315.6	0.75	2314.6	0.93	
Fuc_1 Hex_3 $HexNAc_6$	2326.6	2.95	2326.7	2.38	2326.7	9.37	
$NeuAc_1$ Hex_6 $HexNAc_3$	2390.5	7.70	2390.6	10.90	2390.7	7.30	
Hex_9 $HexNAc_2$	2396.5	100.00	2396.7	100.00	2396.7	100.00	**Relative Intensity**
$NeuAc_1$ Fuc_1 Hex_4 $HexNAc_4$	2402.4	1.76	2402.6	2.37	2401.6	9.16	
Fuc_2 Hex_5 $HexNAc_4$	2418.5	1.32	2417.6	2.00	2417.7	1.97	
$NeuAc_1$ Hex_5 $HexNAc_4$	2431.5	3.43	2431.6	4.89	2431.7	5.65	
Fuc_1 Hex_5 $HexNAc_5$	2489.5	6.20	2489.6	0.94	2489.7	1.21	
Hex_6 $HexNAc_5$	2519.5	2.12	2519.6	0.49	2519.7	0.75	
Fuc_3 Hex_5 $HexNAc_4$	2592.5	1.72	2591.6	1.37	2591.7	1.31	
$NeuAc_1$ Fuc_1 Hex_5 $HexNAc_4$	2605.5	6.23	2605.7	7.62	2605.8	9.61*	*Sialylated product

Composition	MDCK		SIAT1		hCK		Remarks
	Mass (m/z)	%	Mass (m/z)	%	Mass (m/z)	%	
$NeuAc_1 Fuc_1 Hex_5 HexNAc_4$<br>$NeuAc_1 Hex_6 HexNAc_4$	2635.5	7.35	2635.7	5.77	2635.8	3.90	
$NeuAc_1 Fuc_1 Hex_4 HexNAc_5$	2646.5	2.09	2646.7	4.31*	2646.8	8.25*	*Sialylated bisected product
$Fuc_2 Hex_5 HexNAc_5$	2663.5	2.79	2662.7	1.54	2662.7	0.98	
$NeuAc_1 Hex_5 HexNAc_5$	2677.5	1.86	2676.7	2.35	2676.8	2.02	
$Fuc_1 Hex_6 HexNAc_5$	2693.5	6.63	2693.6	0.54	2693.8	0.95	
$Fuc_1 Hex_5 HexNAc_6$	2734.5	0.82	2734.6	0.30	2734.8	1.02	
$Hex_6 HexNAc_6$	2764.1	0.12	2764.4	0.10	2764.8	0.64	
$NeuAc_1 Fuc_2 Hex_5 HexNAc_4$	2779.5	3.67	2778.7	1.99	2778.8	1.37	
$NeuAc_2 Hex_5 HexNAc_4$	2792.4	0.68	2792.7	7.04	2792.8	6.87	
$NeuAc_1 Fuc_1 Hex_6 HexNAc_4$	2809.6	13.34	2809.4	0.28	2809.8	1.98	
$Fuc_3 Hex_5 HexNAc_5$	2837.5	2.33	2836.7	3.34	2836.8	1.23	
$NeuAc_1 Fuc_1 Hex_5 HexNAc_5$	2850.5	4.79	2850.7	5.76	2850.8	4.52	
$Fuc_2 Hex_6 HexNAc_5$	2867.5	8.98	2866.6	0.97	2866.8	0.66	
$NeuAc_1 Hex_6 HexNAc_5$	2880.5	2.72	2880.7	2.40	2880.8	1.96	
$Fuc_1 Hex_6 HexNAc_6$	2938.5	1.70	2938.7	0.86	2938.8	0.67	
$NeuAc_1 Fuc_3 Hex_5 HexNAc_4$	2953.5	0.90	2952.7	1.79	2952.8	1.13	
$NeuAc_2 Fuc_1 Hex_5 HexNAc_4$	2966.5	7.42	2966.7	17.23*	2966.8	22.40*	*Sialylated product
$NeuAc_1 Fuc_2 Hex_5 HexNAc_5$	3024.5	2.20	3023.6	2.14	3024.7	0.87	
$NeuAc_2 Hex_5 HexNAc_5$	3037.5	0.86	3037.6	6.64*	3037.7	2.05	*Sialylated/bisected product
$Fuc_3 Hex_6 HexNAc_5$	3040.5	1.60	3040.5	1.87	3040.7	0.57	
$NeuAc_1 Fuc_1 Hex_6 HexNAc_5$	3054.5	8.16	3054.7	5.55	3054.7	2.78	
$Fuc_2 Hex_6 HexNAc_6$	3112.5	1.44	3112.6	0.76	3112.6	0.31	
$NeuAc_1 Hex_6 HexNAc_6$	3126.4	0.87	3125.6	0.67	3125.7	1.34	
$NeuAc_2 Fuc_2 Hex_5 HexNAc_4$	3140.5	0.80	3140.6	0.54	3140.7	0.49	
$Fuc_1 Hex_7 HexNAc_6$	3142.5	2.75	3142.6	1.18	3142.7	0.79	
$Fuc_1 Hex_6 HexNAc_7$	3183.4	0.43	3183.6	0.73	3183.6	0.23	
$NeuAc_1 Fuc_3 Hex_5 HexNAc_5$	3198.4	0.64	3197.6	3.12	3197.7	0.55	
$NeuAc_2 Fuc_1 Hex_5 HexNAc_5$	3212.4	5.27	3212.6	39.75*	3212.6	11.33*	*Sialylated/bisected product
$Hex_7 HexNAc_7$	3213.4	5.62	3213.8	25.82	3214.0	5.10	
$NeuAc_1 Fuc_2 Hex_6 HexNAc_5$	3228.4	1.67	3227.5	2.42	3227.6	1.18	
$NeuAc_2 Hex_6 HexNAc_5$	3240.4	0.83	3241.6	3.33	3241.7	1.36	
$Fuc_3 Hex_6 HexNAc_6$	3286.4	1.11	3285.5	0.88	3285.6	0.66	
$NeuAc_1 Fuc_1 Hex_6 HexNAc_6$	3299.4	2.64	3299.5	1.14	3299.6	2.69	
$NeuAc_1 Hex_7 HexNAc_7$	3314.5	0.54	3314.7	0.38	3314.7	0.33	
$Fuc_2 Hex_7 HexNAc_6$	3316.4	1.90	3316.7	0.78	3316.7	0.87	

Composition	MDCK		SIAT1		hCK		Remarks
	Mass (m/z)	%	Mass (m/z)	%	Mass (m/z)	%	
$NeuAc_3$ Fuc_2 Hex_5 $HexNAc_4$	3327.4	0.32	3327.6	0.36	3327.7	0.22	
$NeuAc_1$ Fuc_1 Hex_7 $HexNAc_6$	3329.3	1.00	3329.6	0.68	3329.6	1.53	
$NeuAc_2$ Fuc_2 Hex_5 $HexNAc_5$	3385.6	0.27	3385.7	0.34	3385.6	0.18	
Fuc_1 Hex_7 $HexNAc_7$	3387.3	0.67	3387.6	0.89	3387.5	0.27	
$NeuAc_1$ Fuc_3 Hex_6 $HexNAc_5$	3402.4	0.77	3402.0	0.27	3401.6	0.53	
$NeuAc_2$ Fuc_1 Hex_6 $HexNAc_5$	3416.2	1.94	3415.5	4.82*	3415.6	2.86*	*Sialylated product
$NeuAc_1$ Fuc_2 Hex_6 $HexNAc_6$	3473.3	1.36	3472.4	1.12	3473.5	0.49	
$NeuAc_2$ Hex_6 $HexNAc_6$	3486.4	0.62	3486.4	1.69	3486.4	0.27	
$NeuAc_2$ Fuc_4 Hex_5 $HexNAc_4$	3489.3	1.73	3488.9	0.60	3487.5	0.83	
Fuc_3 Hex_7 $HexNAc_6$	3490.3	3.29	3490.4	0.48	3490.4	0.64	
$NeuAc_1$ Fuc_1 Hex_7 $HexNAc_6$	3503.3	3.88	3503.4	1.34	3503.5	2.67	
$NeuAc_2$ Fuc_2 Hex_5 $HexNAc_5$	3559.8	0.12	3559.9	0.09	3560.0	0.04	
Fuc_2 Hex_7 $HexNAc_7$	3561.2	0.56	3561.8	0.14	3561.8	0.13	
$NeuAc_1$ Hex_7 $HexNAc_7$	3574.5	0.26	3574.3	0.43	3574.4	0.26	
$NeuAc_2$ Fuc_2 Hex_6 $HexNAc_5$	3590.2	1.47	3589.4	2.28	3589.4	0.80	
Fuc_1 Hex_8 $HexNAc_7$	3591.8	0.38	3591.8	1.00	3591.9	0.43	
$NeuAc_3$ Hex_6 $HexNAc_5$	3604.2	0.71	3603.0	0.35	3602.4	0.99	
Fuc_1 Hex_7 $HexNAc_8$	3633.0	0.28	3632.3	0.57	3632.4	0.28	
$NeuAc_1$ Fuc_3 Hex_6 $HexNAc_6$	3647.2	0.73	3646.3	2.06	3646.4	0.34	
$NeuAc_2$ Fuc_1 Hex_6 $HexNAc_6$	3661.0	1.93	3661.2	8.78*	3661.3	3.51*	*Sialylated product
Hex_8 $HexNAc_8$	3662.8	0.72	3662.7	3.44	3662.7	2.44	
$NeuAc_1$ Fuc_2 Hex_7 $HexNAc_6$	3677.1	2.68	3676.3	0.91	3676.4	0.57	
$NeuAc_2$ Hex_7 $HexNAc_6$	3691.1	0.99	3690.3	1.45	3690.4	1.30	
Fuc_3 Hex_7 $HexNAc_7$	3736.1	0.55	3735.8	0.19	3736.1	0.13	
$NeuAc_1$ Fuc_1 Hex_7 $HexNAc_7$	3748.0	0.91	3748.2	0.57	3748.3	0.76	
$NeuAc_2$ Fuc_3 Hex_6 $HexNAc_5$	3764.0	0.79	3763.5	0.69	3763.3	0.30	
$NeuAc_3$ Fuc_1 Hex_6 $HexNAc_5$	3776.9	1.55	3777.2	12.24*	3777.2	2.79*	*Sialylated product
Fuc_2 Hex_7 $HexNAc_8$	3807.0	0.57	3806.1	0.60	3807.3	0.33	
$NeuAc_2$ Fuc_2 Hex_6 $HexNAc_6$	3834.0	0.66	3834.2	1.37	3834.2	0.42	
Fuc_1 Hex_8 $HexNAc_8$	3837.0	1.60	3837.1	1.97	3837.1	0.37	
$NeuAc_3$ Hex_6 $HexNAc_6$	3848.0	0.52	3848.1	1.81	3848.1	0.25	
$NeuAc_1$ Fuc_3 Hex_7 $HexNAc_6$	3852.0	1.96	3851.5	1.49	3851.3	1.55	
$NeuAc_2$ Fuc_1 Hex_7 $HexNAc_6$	3865.1	6.38	3865.1	6.43	3865.4	6.71	
Fuc_4 Hex_7 $HexNAc_7$	3910.9	0.93	3910.1	0.58	3910.2	0.30	
$NeuAc_2$ Hex_7 $HexNAc_7$	3937.8	0.66	3937.1	0.33	3937.1	0.23	
$NeuAc_2$ Fuc_4 Hex_6 $HexNAc_5$	3938.0	0.85	3938.2	0.77	3938.4	0.31	

Composition	MDCK Mass (m/z)	MDCK %	SIAT1 Mass (m/z)	SIAT1 %	hCK Mass (m/z)	hCK %	Remarks
Fuc_3 Hex_8 $HexNAc_7$	3940.0	1.64	3940.1	0.89	3940.2	0.50	
$NeuAc_3$ Fuc_2 Hex_6 $HexNAc_5$	3951.9	1.42	3951.0	0.52	3951.1	0.13	
$NeuAc_1$ Fuc_1 Hex_8 $HexNAc_7$	3953.1	2.06	3953.3	0.88	3953.2	1.51	
$NeuAc_2$ Fuc_3 Hex_6 $HexNAc_6$	4007.8	0.40	4009.0	2.36	4009.1	0.33	
Fuc_2 Hex_8 $HexNAc_8$	4011.1	0.37	4011.4	0.47	4011.3	0.21	
$NeuAc_3$ Fuc_1 Hex_6 $HexNAc_6$	4022.1	0.70	4022.0	18.94*	4022.2	1.82*	*Sialylated bisected product
$NeuAc_1$ Hex_8 $HexNAc_8$	4024.3	0.37	4024.3	8.35	4024.3	1.20	
$NeuAc_1$ Fuc_4 Hex_7 $HexNAc_6$	4026.1	0.53	4026.0	5.34	4026.0	0.70	
$NeuAc_2$ Fuc_2 Hex_7 $HexNAc_6$	4039.3	0.28	4039.1	1.64	4039.1	1.31	
$NeuAc_3$ Hex_7 $HexNAc_6$	4052.7	0.59	4051.9	2.11	4051.1	1.52	
$NeuAc_3$ Fuc_3 Hex_6 $HexNAc_5$	4125.7	1.13	4124.8	0.31	4124.9	0.20	
$NeuAc_1$ Fuc_2 Hex_8 $HexNAc_7$	4127.0	0.99	4127.1	0.41	4127.0	0.43	
$NeuAc_4$ Fuc_1 Hex_6 $HexNAc_5$	4138.5	0.25	4138.8	0.39	4138.9	0.27	
$NeuAc_2$ Hex_8 $HexNAc_7$	4141.6	0.72	4140.2	0.30	4140.3	0.17	
$NeuAc_2$ Fuc_4 Hex_6 $HexNAc_6$	4183.4	0.23	4183.6	0.14	4183.6	0.05	
Fuc_3 Hex_8 $HexNAc_8$	4184.6	0.33	4185.2	0.11	4184.9	0.17	
$NeuAc_1$ Fuc_1 Hex_8 $HexNAc_8$	4197.6	0.34	4197.9	0.55	4197.6	0.05	
$NeuAc_2$ Fuc_3 Hex_7 $HexNAc_6$	4211.6	0.47	4211.9	0.64	4210.8	0.22	
$NeuAc_3$ Fuc_1 Hex_7 $HexNAc_6$	4225.8	2.27	4225.9	2.67	4225.9	3.97*	*Sialylated product
$NeuAc_2$ Fuc_2 Hex_7 $HexNAc_7$	4284.3	0.55	4284.6	0.52	4284.6	0.16	
Fuc_1 Hex_9 $HexNAc_9$	4286.4	0.80	4286.1	0.19	4286.1	0.12	
$NeuAc_3$ Hex_7 $HexNAc_7$	4298.4	0.58	4297.6	0.42	4297.7	0.17	
$NeuAc_3$ Fuc_4 Hex_6 $HexNAc_5$	4299.2	0.28	4299.2	0.18	4299.2	0.08	
$NeuAc_1$ Fuc_3 Hex_8 $HexNAc_7$	4301.5	1.44	4301.2	0.19	4301.2	0.11	
$NeuAc_2$ Fuc_1 Hex_8 $HexNAc_7$	4314.2	1.41	4314.4	1.41	4313.8	1.77	
$NeuAc_2$ Hex_8 $HexNAc_8$	4385.3	0.22	4385.2	0.07	4385.2	0.04	
$NeuAc_2$ Fuc_4 Hex_7 $HexNAc_6$	4387.6	0.28	4387.2	0.08	4387.2	0.04	
Fuc_3 Hex_9 $HexNAc_8$	4389.2	0.31	4389.2	0.06	4389.2	0.04	
$NeuAc_3$ Fuc_2 Hex_7 $HexNAc_6$	4399.3	0.51	4399.7	0.30	4399.8	0.21	
$NeuAc_1$ Fuc_1 Hex_9 $HexNAc_8$	4402.5	0.93	4402.4	0.50	4402.4	0.17	
$NeuAc_4$ Hex_7 $HexNAc_6$	4413.2	0.87	4412.4	0.28	4412.4	0.31	
$NeuAc_3$ Fuc_1 Hex_7 $HexNAc_7$	4471.4	1.30	4471.2	1.29	4471.2	0.11	
$NeuAc_1$ Hex_9 $HexNAc_9$	4473.5	0.69	4473.5	1.60	4473.5	0.40	
$NeuAc_1$ Fuc_4 Hex_8 $HexNAc_7$	4475.4	0.44	4475.6	0.38	4475.6	0.14	
$NeuAc_2$ Fuc_2 Hex_8 $HexNAc_7$	4486.2	0.60	4487.6	0.39	4487.5	0.37	
$NeuAc_3$ Hex_8 $HexNAc_7$	4501.1	0.32	4500.3	0.28	4500.3	0.25	

Composition	MDCK		SIAT1		hCK		Remarks
	Mass (m/z)	%	Mass (m/z)	%	Mass (m/z)	%	
NeuAc$_3$ Fuc$_3$ Hex$_7$ HexNAc$_6$	4573.0	0.31	4574.2	0.33	4574.2	0.09	
NeuAc$_1$ Fuc$_2$ Hex$_9$ HexNAc$_8$	4576.6	0.23	4576.3	0.26	4576.3	0.13	
NeuAc$_4$ Fuc$_1$ Hex$_7$ HexNAc$_6$	4587.3	3.04	4587.4	1.98	4587.4	1.64	
NeuAc$_2$ Fuc$_4$ Hex$_7$ HexNAc$_7$	4632.3	0.11	4632.4	0.09	4632.4	0.05	
Fuc$_3$ Hex$_9$ HexNAc$_9$	4634.4	0.10	4634.2	0.14	4634.2	0.05	
NeuAc$_3$ Fuc$_2$ Hex$_7$ HexNAc$_7$	4645.7	0.16	4645.8	0.06	4645.8	0.02	
NeuAc$_1$ Fuc$_1$ Hex$_9$ HexNAc$_9$	4647.7	0.13	4647.2	0.22	4647.2	0.06	
NeuAc$_1$ Fuc$_5$ Hex$_8$ HexNAc$_7$	4649.5	0.08	4649.2	0.12	4649.2	0.04	
NeuAc$_2$ Fuc$_3$ Hex$_8$ HexNAc$_7$	4662.5	0.13	4662.3	0.34	4662.3	0.04	
NeuAc$_3$ Fuc$_1$ Hex$_8$ HexNAc$_7$	4675.1	1.74	4675.1	3.68*	4675.1	2.00	*Sialylated product
NeuAc$_1$ Fuc$_4$ Hex$_8$ HexNAc$_8$	4719.8	0.27	4719.9	0.16	4720.1	0.06	
NeuAc$_2$ Fuc$_2$ Hex$_8$ HexNAc$_8$	4733.6	0.17	4733.4	0.06	4733.4	0.03	
Fuc$_1$ Hex$_{10}$ HexNAc$_{10}$	4735.5	0.09	4735.4	0.07	4735.4	0.03	
NeuAc$_3$ Hex$_8$ HexNAc$_8$	4746.7	0.17	4746.4	0.08	4746.4	0.03	
NeuAc$_3$ Fuc$_4$ Hex$_7$ HexNAc$_6$	4748.5	0.15	4748.4	0.10	4748.4	0.04	
NeuAc$_1$ Fuc$_3$ Hex$_9$ HexNAc$_8$	4750.5	0.17	4750.4	0.10	4750.4	0.04	
NeuAc$_4$ Fuc$_2$ Hex$_7$ HexNAc$_6$	4760.7	0.31	4760.9	0.23	4761.0	0.11	
NeuAc$_2$ Fuc$_1$ Hex$_9$ HexNAc$_8$	4763.5	0.39	4763.5	0.12	4763.5	0.08	
NeuAc$_2$ Fuc$_5$ Hex$_7$ HexNAc$_7$	4806.6	0.10	4806.4	0.04	4806.4	0.02	
Fuc$_4$ Hex$_9$ HexNAc$_9$	4808.6	0.30	4808.4	0.04	4808.4	0.02	
NeuAc$_3$ Fuc$_3$ Hex$_7$ HexNAc$_7$	4819.5	0.10	4819.4	0.04	4819.4	0.01	
NeuAc$_1$ Fuc$_2$ Hex$_9$ HexNAc$_9$	4821.5	0.14	4821.4	0.05	4821.4	0.02	
NeuAc$_1$ Fuc$_6$ Hex$_8$ HexNAc$_7$	4823.7	0.11	4823.4	0.04	4823.4	0.02	
NeuAc$_2$ Hex$_9$ HexNAc$_9$	4834.5	0.35	4834.6	0.24	4834.6	0.03	
NeuAc$_2$ Fuc$_4$ Hex$_8$ HexNAc$_7$	4836.6	0.21	4836.4	0.07	4836.4	0.03	
NeuAc$_3$ Fuc$_2$ Hex$_8$ HexNAc$_7$	4849.1	0.11	4849.4	0.06	4849.4	0.03	
NeuAc$_1$ Fuc$_1$ Hex$_{10}$ HexNAc$_9$	4851.6	0.34	4851.4	0.07	4851.4	0.03	
NeuAc$_4$ Hex$_8$ HexNAc$_7$	4862.6	0.21	4861.8	0.25	4861.8	0.10	
NeuAc$_1$ Fuc$_5$ Hex$_8$ HexNAc$_8$	4894.6	0.10	4894.5	0.06	4894.5	0.02	
NeuAc$_2$ Fuc$_3$ Hex$_8$ HexNAc$_8$	4907.6	0.14	4907.5	0.12	4907.5	0.02	
Fuc$_2$ Hex$_{10}$ HexNAc$_{10}$	4909.5	0.13	4909.5	0.07	4909.5	0.02	
NeuAc$_3$ Fuc$_1$ Hex$_8$ HexNAc$_8$	4920.7	0.20	4920.5	0.50	4920.5	0.05	
NeuAc$_1$ Hex$_{10}$ HexNAc$_{10}$	4922.5	0.29	4922.5	0.28	4922.5	0.04	
NeuAc$_1$ Fuc$_4$ Hex$_9$ HexNAc$_8$	4924.5	0.18	4924.6	0.13	4924.6	0.04	
NeuAc$_2$ Fuc$_2$ Hex$_9$ HexNAc$_8$	4937.7	0.35	4937.5	0.24	4937.6	0.12	
NeuAc$_5$ Fuc$_1$ Hex$_7$ HexNAc$_6$	4948.4	0.12	4947.5	0.09	4947.5	0.03	

Composition	MDCK		SIAT1		hCK		Remarks
	Mass (m/z)	%	Mass (m/z)	%	Mass (m/z)	%	
$NeuAc_3 Hex_9 HexNAc_8$	4950.6	0.14	4950.5	0.24	4950.5	0.12	
$NeuAc_2 Fuc_6 Hex_7 HexNAc_7$	4980.5	0.07	4980.5	0.09	4980.5	0.02	
$NeuAc_3 Fuc_4 Hex_7 HexNAc_7$	4993.5	0.06	4993.4	0.08	4993.5	0.02	
$NeuAc_1 Fuc_3 Hex_9 HexNAc_9$	4995.3	0.15	4995.5	0.08	4995.5	0.03	
$NeuAc_2 Fuc_1 Hex_9 HexNAc_9$	5008.8	0.05	5008.5	0.13	5008.7	0.04	
$NeuAc_2 Fuc_5 Hex_8 HexNAc_7$	5010.6	0.06	5010.5	0.07	5010.5	0.03	
$NeuAc_3 Fuc_3 Hex_8 HexNAc_7$	5023.5	0.10	5023.7	0.06	5023.6	0.03	
$NeuAc_1 Fuc_2 Hex_{10} HexNAc_9$	5025.6	0.10	5025.6	0.08	5025.6	0.03	
$NeuAc_4 Fuc_1 Hex_8 HexNAc_7$	5036.7	0.17	5036.3	0.86*	5036.3	0.08	*Sialylated product
$NeuAc_1 Fuc_6 Hex_8 HexNAc_8$	5068.6	0.04	5068.4	0.11	5068.4	0.03	
$NeuAc_2 Fuc_4 Hex_8 HexNAc_8$	5081.6	0.04	5081.6	0.05	5081.6	0.02	
$Fuc_3 Hex_{10} HexNAc_{10}$	5083.5	0.05	5083.6	0.05	5083.6	0.02	
$NeuAc_3 Fuc_2 Hex_8 HexNAc_8$	5094.8	0.05	5094.6	0.06	5094.6	0.02	
$NeuAc_1 Fuc_1 Hex_{10} HexNAc_{10}$	5096.8	0.05	5096.5	0.07	5096.6	0.02	
$NeuAc_1 Fuc_5 Hex_9 HexNAc_8$	5098.6	0.04	5098.7	0.03	5098.6	0.01	
$NeuAc_2 Fuc_3 Hex_9 HexNAc_8$	5111.3	0.14	5111.4	0.18	5111.4	0.05	
$NeuAc_3 Fuc_1 Hex_9 HexNAc_8$	5124.8	0.34	5125.2	1.22*	5125.2	0.46	*Sialylated product
$NeuAc_1 Fuc_4 Hex_9 HexNAc_9$	5169.7	0.17	5169.8	0.03	5169.8	0.02	
$NeuAc_2 Fuc_2 Hex_9 HexNAc_9$	5182.9	0.09	5182.6	0.03	5182.6	0.01	
$NeuAc_2 Fuc_6 Hex_8 HexNAc_7$	5184.8	0.06	5184.7	0.02	5184.7	0.01	
$NeuAc_3 Hex_9 HexNAc_9$	5195.8	0.08	5195.5	0.03	5195.5	0.02	
$NeuAc_3 Fuc_4 Hex_8 HexNAc_7$	5197.7	0.09	5197.9	0.05	5197.8	0.01	
$NeuAc_1 Fuc_3 Hex_{10} HexNAc_9$	5199.7	0.08	5199.7	0.03	5199.6	0.02	
$NeuAc_4 Fuc_2 Hex_8 HexNAc_7$	5210.9	0.21	5210.7	0.04	5210.7	0.02	
$NeuAc_2 Fuc_1 Hex_{10} HexNAc_9$	5212.7	0.16	5212.6	0.04	5212.6	0.03	
$NeuAc_2 Fuc_5 Hex_8 HexNAc_8$	5255.6	0.06	5255.6	0.02	5255.6	0.01	
$NeuAc_3 Fuc_3 Hex_8 HexNAc_8$	5268.7	0.05	5268.7	0.03	5268.7	0.01	
$NeuAc_1 Fuc_2 Hex_{10} HexNAc_{10}$	5270.5	0.04	5270.7	0.03	5270.7	0.02	
$NeuAc_1 Fuc_6 Hex_9 HexNAc_8$	5272.8	0.04	5272.8	0.02	5272.8	< 0.01	
$NeuAc_2 Hex_{10} HexNAc_{10}$	5283.7	0.08	5283.8	0.08	5283.8	0.02	
$NeuAc_2 Fuc_4 Hex_9 HexNAc_8$	5285.8	0.06	5285.8	0.04	5285.8	0.01	
$NeuAc_3 Fuc_2 Hex_9 HexNAc_8$	5298.8	0.17	5298.8	0.19	5298.8	0.06	
$NeuAc_1 Fuc_1 Hex_{11} HexNAc_{10}$	5300.8	0.08	5300.8	0.07	5300.8	0.02	
$NeuAc_4 Hex_9 HexNAc_8$	5311.8	0.07	5311.8	0.24	5311.8	0.06	
$NeuAc_1 Fuc_5 Hex_9 HexNAc_9$	5343.8	0.04	5343.8	0.04	5343.8	0.02	
$NeuAc_2 Fuc_3 Hex_9 HexNAc_9$	5356.8	0.04	5356.8	0.05	5356.8	0.02	

Composition	MDCK		SIAT1		hCK		Remarks
	Mass (m/z)	%	Mass (m/z)	%	Mass (m/z)	%	
$Fuc_2 Hex_{11} HexNAc_{11}$	5358.8	0.03	5358.6	0.03	5358.6	0.01	
$NeuAc_3 Fuc_1 Hex_9 HexNAc_9$	5369.8	0.04	5369.8	0.12	5369.8	0.03	
$NeuAc_1 Hex_{11} HexNAc_{11}$	5371.8	0.04	5371.8	0.06	5371.8	0.02	
$NeuAc_1 Fuc_4 Hex_{10} HexNAc_9$	5373.8	0.03	5373.8	0.03	5373.8	0.02	
$NeuAc_4 Fuc_3 Hex_8 HexNAc_7$	5384.8	0.09	5384.8	0.07	5384.8	0.03	
$NeuAc_2 Fuc_2 Hex_{10} HexNAc_9$	5386.8	0.07	5386.8	0.04	5386.8	0.03	
$NeuAc_5 Fuc_1 Hex_8 HexNAc_7$	5397.8	0.04	5397.8	0.07	5397.8	0.04	
$NeuAc_3 Hex_{10} HexNAc_9$	5399.8	0.04	5399.8	0.07	5399.8	0.04	
$NeuAc_2 Fuc_6 Hex_8 HexNAc_8$	5429.8	0.03	5429.8	0.04	5429.8	0.02	
$NeuAc_3 Fuc_4 Hex_8 HexNAc_8$	5442.8	0.03	5442.8	0.03	5442.8	0.01	
$NeuAc_1 Fuc_3 Hex_{10} HexNAc_{10}$	5444.8	0.03	5444.8	0.03	5444.8	0.01	
$NeuAc_1 Fuc_7 Hex_9 HexNAc_8$	5446.8	0.02	5446.8	0.02	5446.8	< 0.01	
$NeuAc_2 Fuc_1 Hex_{10} HexNAc_{10}$	5457.8	0.02	5457.8	0.03	5457.8	0.01	
$NeuAc_2 Fuc_5 Hex_9 HexNAc_8$	5459.8	0.02	5459.8	0.02	5459.8	0.01	
$NeuAc_3 Fuc_3 Hex_9 HexNAc_8$	5472.8	0.03	5472.8	0.05	5472.8	0.02	
$NeuAc_1 Fuc_2 Hex_{11} HexNAc_{10}$	5474.8	0.03	5474.8	0.03	5474.8	0.01	
$NeuAc_4 Fuc_1 Hex_9 HexNAc_8$	5487.1	0.06	5487.4	0.34	5487.4	0.05	
$NeuAc_2 Fuc_4 Hex_9 HexNAc_9$	5530.9	0.02	5530.7	0.03	5530.6	0.02	
$Fuc_3 Hex_{11} HexNAc_{11}$	5532.9	0.02	5532.8	0.01	5532.8	0.01	
$NeuAc_1 Fuc_1 Hex_{11} HexNAc_{11}$	5545.9	0.02	5545.9	0.02	5545.8	< 0.01	
$NeuAc_1 Fuc_5 Hex_{10} HexNAc_9$	5547.9	0.02	5547.9	0.01	5547.9	< 0.01	
$NeuAc_4 Fuc_4 Hex_8 HexNAc_7$	5558.9	0.05	5558.9	0.03	5558.8	0.01	
$NeuAc_2 Fuc_3 Hex_{10} HexNAc_9$	5560.9	0.04	5560.9	0.03	5560.9	0.01	
$NeuAc_3 Fuc_1 Hex_{10} HexNAc_9$	5574.4	0.02	5574.4	0.06	5574.4	0.08	
$NeuAc_1 Fuc_4 Hex_{10} HexNAc_{10}$	5618.9	0.09	5618.9	0.01	5618.8	< 0.01	
$NeuAc_2 Fuc_2 Hex_{10} HexNAc_{10}$	5631.9	0.03	5631.9	0.01	5631.8	< 0.01	
$NeuAc_2 Fuc_6 Hex_9 HexNAc_8$	5633.9	0.02	5633.9	0.01	5633.9	< 0.01	
$NeuAc_3 Fuc_4 Hex_9 HexNAc_8$	5646.9	0.04	5646.9	0.02	5646.9	0.01	
$NeuAc_1 Fuc_3 Hex_{11} HexNAc_{10}$	5648.9	0.03	5648.9	0.02	5648.9	< 0.01	
$NeuAc_4 Fuc_2 Hex_9 HexNAc_8$	5659.9	0.06	5659.9	0.04	5659.9	0.02	
$NeuAc_2 Fuc_1 Hex_{11} HexNAc_{10}$	5661.9	0.04	5661.8	0.02	5661.8	0.01	
$NeuAc_5 Hex_9 HexNAc_8$	5673.1	0.01	5673.2	0.02	5673.2	< 0.01	
$NeuAc_1 Fuc_2 Hex_{11} HexNAc_{11}$	5720.2	0.02	5720.1	0.02	5720.1	0.01	
$NeuAc_1 Fuc_6 Hex_{10} HexNAc_9$	5722.1	0.01	5722.0	0.02	5722.0	0.01	
$NeuAc_2 Hex_{11} HexNAc_{11}$	5733.1	0.02	5733.0	0.03	5733.0	0.01	
$NeuAc_2 Fuc_4 Hex_{10} HexNAc_9$	5735.1	0.02	5735.2	0.01	5735.2	0.01	

Composition	MDCK		SIAT1		hCK		Remarks
	Mass (m/z)	%	Mass (m/z)	%	Mass (m/z)	%	
$NeuAc_3 Fuc_2 Hex_{10} HexNAc_9$	5748.1	0.03	5748.0	0.04	5748.0	0.02	
$NeuAc_1 Fuc_1 Hex_{12} HexNAc_{11}$	5750.1	0.02	5750.2	0.01	5750.2	0.01	
$NeuAc_4 Hex_{10} HexNAc_9$	5761.2	0.02	5761.0	0.03	5761.0	0.02	
$NeuAc_3 Fuc_1 Hex_{10} HexNAc_{10}$	5819.1	0.02	5819.0	0.02	5819.0	< 0.01	
$NeuAc_1 Hex_{12} HexNAc_{12}$	5821.1	0.02	5821.0	0.02	5821.0	< 0.01	
$NeuAc_1 Fuc_4 Hex_{11} HexNAc_{10}$	5823.1	0.01	5823.0	0.01	5823.0	< 0.01	
$NeuAc_4 Fuc_3 Hex_9 HexNAc_8$	5834.0	0.02	5834.0	0.02	5834.0	0.01	
$NeuAc_2 Fuc_2 Hex_{11} HexNAc_{10}$	5836.0	0.02	5836.0	0.02	5836.0	0.01	
$Fuc_1 Hex_{13} HexNAc_{12}$	5838.0	0.01	5838.3	0.01	5838.0	< 0.01	
$NeuAc_5 Fuc_1 Hex_9 HexNAc_8$	5847.2	0.03	5847.0	0.02	5847.0	< 0.01	
$NeuAc_1 Fuc_3 Hex_{11} HexNAc_{11}$	5894.1	0.02	5894.0	0.01	5894.0	< 0.01	
$NeuAc_2 Fuc_1 Hex_{11} HexNAc_{11}$	5907.1	0.02	5907.0	0.01	5907.0	< 0.01	
$NeuAc_2 Fuc_5 Hex_{10} HexNAc_9$	5909.1	0.02	5909.1	0.01	5909.1	< 0.01	
$NeuAc_3 Fuc_3 Hex_{10} HexNAc_9$	5922.1	0.02	5922.1	0.02	5922.1	0.01	
$NeuAc_1 Fuc_2 Hex_{12} HexNAc_{11}$	5924.1	0.02	5924.1	0.01	5924.1	< 0.01	
$NeuAc_4 Fuc_1 Hex_{10} HexNAc_9$	5935.1	0.03	5935.4	0.10	5935.4	0.04	
$NeuAc_1 Fuc_6 Hex_{10} HexNAc_{10}$	5967.1	0.02	5967.1	0.02	5967.1	< 0.01	
$NeuAc_1 Fuc_1 Hex_{12} HexNAc_{12}$	5995.2	0.01	5995.2	0.01	5995.2	< 0.01	
$NeuAc_1 Fuc_5 Hex_{11} HexNAc_{10}$	5997.2	0.01	5997.2	< 0.01	5997.2	< 0.01	
