# Supplementary material for: Sialylated and sulfated N-Glycans in MDCK and engineered MDCK cells for influenza virus studies
Source: Sci Rep. 2022 Jul 26;12:12757. doi: 10.1038/s41598-022-16605-5 (PMC9325728; doi:10.1038/s41598-022-16605-5)
Supplement: Supplementary file 2 — Supplementary Table S2. [file 41598_2022_16605_MOESM2_ESM.pdf]

Supplementary Table 2

Percentage values of structural features (%)

Sialylation (%)

	MDCK	SIAT1	hCK
non-sialylated	73.87	60.17	70.40
sialylated	26.13	39.83	29.60
1-sia	16.35	15.23	16.71
2-sia	6.17	16.33	9.95
3-sia	2.75	7.52	2.51
4-sia	0.83	0.72	0.41
5-sia	0.03	0.03	0.01

Fucosylation (%)

	MDCK	SIAT1	hCK
non-fucosylated	66.59	62.99	67.51
fucosylated	33.41	37.01	32.49
1-fuc	21.03	27.55	27.65
2-fuc	7.38	4.24	2.66
3-fuc	3.75	3.77	1.69
4-fuc	1.10	1.33	0.43
5-fuc	0.08	0.06	0.03
6-fuc	0.06	0.05	0.02
7-fuc	< 0.01	< 0.01	< 0.01

Bisection (%)

	MDCK	SIAT1	hCK
non-bisected	95.68	90.88	94.75
bisected	4.32	9.12	5.24
sia/fuc	1.54	6.67	2.37
sia/non-fuc	0.13	0.97	0.31
non-sia/fuc	2.65	1.48	2.57
non-sia/non-fuc	0	0	0
