# Supplementary material for: Sialylated and sulfated N-Glycans in MDCK and engineered MDCK cells for influenza virus studies
Source: Sci Rep. 2022 Jul 26;12:12757. doi: 10.1038/s41598-022-16605-5 (PMC9325728; doi:10.1038/s41598-022-16605-5)
Supplement: Supplementary file 3 — Supplementary Table S3. [file 41598_2022_16605_MOESM3_ESM.pdf]

Supplementary Table 3

List of representative peaks of N-glycans by MALDI-TOF-MS analysis in MDCK cell line

Composition	Mass (m/z)	MDCK Non %	MDCK NeuA %	MDCK NeuS %	Remarks
Hex_5 $HexNAc_2$	1580	50.03	31.46	34.12	
Hex_6 $HexNAc_2$	1784	57.73	44.64	41.02	
Fuc_1 Hex_3 $HexNAc_4$	1836	8.11	5.95	4.41	
Hex_7 $HexNAc_2$	1988	65.65	58.32	51.89	
Hex_6 $HexNAc_3$	2029	1.50	10.61	3.63	
Hex_5 $HexNAc_4$	2070	5.64	16.66	8.65	
Fuc_1 Hex_3 $HexNAc_5$	2081	7.00	1.63	2.81	
Hex_8 $HexNAc_2$	2192	93.62	90.09	87.88	
Fuc_1 Hex_5 $HexNAc_4$	2244	5.61	35.74*	17.11*	*Desialylated product
Hex_9 $HexNAc_2$	2396	100.00	100.00	100.00	**Relative intensity**
Fuc_1 Hex_6 $HexNAc_4$	2448	2.87	5.97	4.02	
Fuc_1 Hex_5 $HexNAc_5$	2489	6.20	24.64*	9.44*	*Desialylated product
Hex_6 $HexNAc_5$	2519	2.12	10.32	3.14	
$NeuAc_1$ Fuc_1 Hex_5 $HexNAc_4$	2605	6.23	0.05**	3.72	**α2,6-Sia
$NeuAc_1$ Fuc_1 Hex_5 $HexNAc_4$<br>$NeuAc_1$ Hex_6 $HexNAc_4$	2635	7.35	0.07	1.49	
Fuc_1 Hex_6 $HexNAc_5$	2693	6.63	42.03*	16.83*	*Desialylated product
$NeuAc_1$ Fuc_1 Hex_6 $HexNAc_4$	2809	13.34	0.03**	3.80	**α2,6-Sia
Fuc_2 Hex_6 $HexNAc_5$	2867	8.98	10.51	6.94	
Fuc_1 Hex_7 $HexNAc_5$	2897	1.25	12.7*	5.98*	*Desialylated product
Fuc_1 Hex_6 $HexNAc_6$	2938	1.70	24.30*	11.87*	*Desialylated product
$NeuAc_2$ Fuc_1 Hex_5 $HexNAc_4$	2966	7.42	0.03	0.17**	**α2,3- and α2,6-Sia
Hex_7 $HexNAc_6$	2968	1.11	9.27	4.32	
$NeuAc_1$ Fuc_1 Hex_6 $HexNAc_5$	3054	8.16	0.04**	5.32	**α2,6-Sia
Fuc_1 Hex_7 $HexNAc_6$	3143	2.75	82.28*	55.00*	*Desialylated product
$NeuAc_2$ Fuc_1 Hex_5 $HexNAc_5$	3212	5.27	0.06	0.06	
$NeuAc_1$ Fuc_1 Hex_6 $HexNAc_6$	3299	2.64	0.04	0.25	
Fuc_2 Hex_7 $HexNAc_6$	3316	1.90	9.88	6.80	
Fuc_1 Hex_8 $HexNAc_6$	3347	4.40	28.02*	10.86*	*Desialylated product
Fuc_1 Hex_7 $HexNAc_7$	3388	0.67	19.02*	8.35*	*Desialylated product
$NeuAc_2$ Fuc_1 Hex_6 $HexNAc_5$	3416	1.94	0.06	0.15	
$NeuAc_1$ Fuc_1 Hex_7 $HexNAc_6$	3503	3.88	0.05**	5.59	**α2,6-Sia
Fuc_1 Hex_8 $HexNAc_7$	3592	0.38	27.48*	9.14*	*Desialylated product
$NeuAc_2$ Fuc_1 Hex_6 $HexNAc_6$	3661	1.93	0.15	0.05	
$NeuAc_1$ Fuc_1 Hex_8 $HexNAc_6$	3708	2.46	0.08**	2.04	**α2,6-Sia
Fuc_2 Hex_8 $HexNAc_7$	3766	1.91	10.91	5.59	
Fuc_1 Hex_9 $HexNAc_7$	3796	2.34	18.28*	6.05	*Desialylated product
$NeuAc_2$ Fuc_1 Hex_7 $HexNAc_6$	3865	6.38	0.51	0.04**	**α2,3- and α2,6-Sia
$NeuAc_1$ Fuc_1 Hex_8 $HexNAc_7$	3953	2.06	0.10	0.53	
Fuc_2 Hex_9 $HexNAc_7$	3970	1.36	5.86	3.45	
Fuc_1 Hex_{10} $HexNAc_7$	4000	0.68	3.31	0.59	
Fuc_1 Hex_9 $HexNAc_8$	4041	0.91	8.61*	2.33*	*Desialylated product

Supplementary Table 3

List of representative peaks of N-glycans by MALDI-TOF-MS analysis in MDCK cell line

Composition	Mass (m/z)	MDCK<br>Non<br>%	 NeuA<br>%	 NeuS<br>%	Remarks
$NeuAc_1$ Fuc_1 Hex_9 $HexNAc_7$	4157	1.41	0.05	0.88	
Fuc_2 Hex_9 $HexNAc_8$	4215	0.73	3.83	1.22	
$NeuAc_3$ Fuc_1 Hex_7 $HexNAc_6$	4226	2.27	0.03**	0.12**	**α2,3- and α2,6-Sia
Fuc_1 Hex_{10} $HexNAc_8$	4246	0.27	2.05	1.54	
$NeuAc_1$ Fuc_1 Hex_9 $HexNAc_8$	4403	0.93	0.19	0.53	
Fuc_2 Hex_{10} $HexNAc_8$	4420	0.42	0.75	0.67	
Fuc_1 Hex_{10} $HexNAc_9$	4491	0.56	1.13	0.54	
$NeuAc_4$ Fuc_1 Hex_7 $HexNAc_6$	4587	3.04	0.02**	0.04**	**α2,3- and α2,6-Sia
$NeuAc_1$ Fuc_1 Hex_{10} $HexNAc_8$	4607	0.56	0.03	0.52	
Fuc_2 Hex_{10} $HexNAc_9$	4665	0.10	0.36	0.05	
$NeuAc_3$ Fuc_1 Hex_8 $HexNAc_7$	4675	1.74	0.02	0.05	
Fuc_1 Hex_{11} $HexNAc_9$	4696	0.07	0.38	0.16	
Fuc_1 Hex_{11} $HexNAc_{10}$	4939	0.24	0.14	0.08	
Fuc_1 Hex_{12} $HexNAc_{10}$	5145	0.05	0.04	0.05	
Fuc_1 Hex_{12} $HexNAc_{11}$	5388	0.09	0.09	0.03	
Fuc_1 Hex_{13} $HexNAc_{11}$	5595	0.01	0.02	0.02	
