# Supplementary material for: Sialylated and sulfated N-Glycans in MDCK and engineered MDCK cells for influenza virus studies
Source: Sci Rep. 2022 Jul 26;12:12757. doi: 10.1038/s41598-022-16605-5 (PMC9325728; doi:10.1038/s41598-022-16605-5)
Supplement: Supplementary file 4 — Supplementary Table S4. [file 41598_2022_16605_MOESM4_ESM.pdf]

Supplementary Table 4

List of representative peaks of N-glycans by MALDI-TOF-MS analysis in SIAT1 cell line

Composition	Mass (m/z)	SIAT1 Non %	SIAT1 NeuA %	SIAT1 NeuS %	Remarks
Hex_5 $HexNAc_2$	1580	20.96	14.32	6.98	
Hex_6 $HexNAc_2$	1784	55.89	49.40	27.54	
Fuc_1 Hex_3 $HexNAc_4$	1836	5.51	0.14	2.00	
Hex_7 $HexNAc_2$	1988	52.65	40.21	27.55	
Hex_6 $HexNAc_3$	2029	0.72	11.36	0.53	
Hex_5 $HexNAc_4$	2070	5.26	23.93*	13.26*	*Product from 2792
Fuc_1 Hex_3 $HexNAc_5$	2081	5.37	0.68	1.68	
Hex_8 $HexNAc_2$	2192	88.87	84.60	58.15	
Fuc_1 Hex_5 $HexNAc_4$	2244	4.60	59.77*	17.16	*Product from 2966
Hex_5 $HexNAc_5$	2315	0.75	15.23*	0.31	*Product from 3037
Hex_9 $HexNAc_2$	2396	100.00	100.00	100.00	**Relative intensity**
Fuc_1 Hex_5 $HexNAc_5$	2489	0.94	68.16*	0.57	*Product from 2850/3212
Hex_6 $HexNAc_5$	2519	0.49	13.14	0.32	
Hex_{10} $HexNAc_2$	2600	0.73	2.62	5.42	
$NeuAc_1$ Fuc_1 Hex_5 $HexNAc_4$	2605	7.62	0.10	1.82	
$NeuAc_1$ Fuc_1 Hex_5 $HexNAc_4$<br>$NeuAc_1$ Hex_6 $HexNAc_4$	2635	5.77	0.12	1.59	
Fuc_1 Hex_6 $HexNAc_5$	2693	0.54	49.19*	0.92	*Product from 3777
Hex_6 $HexNAc_6$	2764	0.10	13.82	0.11	
$NeuAc_2$ Hex_5 $HexNAc_4$	2792	7.04	0.18	1.34	
$NeuAc_1$ Fuc_1 Hex_5 $HexNAc_5$	2850	5.76	0.14**	3.44	**α2,6-Sia
Fuc_1 Hex_6 $HexNAc_6$	2938	0.86	88.49*	0.41	*Product from 3661/4022
$NeuAc_2$ Fuc_1 Hex_5 $HexNAc_4$	2966	17.23	0.11**	14.61	**α2,6-Sia
Hex_7 $HexNAc_6$	2968	2.20	4.64	6.47	
$NeuAc_2$ Hex_5 $HexNAc_5$	3037	6.64	0.15	2.75	
$NeuAc_1$ Fuc_1 Hex_6 $HexNAc_5$	3054	5.55	0.10**	4.37	**α2,6-Sia
Fuc_1 Hex_7 $HexNAc_6$	3143	1.18	32.64*	0.96	*Product from 3504/3865
$NeuAc_2$ Fuc_1 Hex_5 $HexNAc_5$	3212	39.75	0.12**	31.28	**α2,6-Sia
$NeuAc_1$ Fuc_1 Hex_6 $HexNAc_6$	3300	1.14	0.13	9.04	
Fuc_2 Hex_7 $HexNAc_6$	3316	0.78	1.27	0.72	
Fuc_1 Hex_8 $HexNAc_6$	3347	0.26	2.89	0.31	
Fuc_1 Hex_7 $HexNAc_7$	3388	0.89	15.64	0.34	
$NeuAc_2$ Fuc_1 Hex_6 $HexNAc_5$	3416	4.82	0.11	15.91*	*Product from 3777
Hex_8 $HexNAc_7$	3418	4.00	1.01	2.33	and α2,6-Sia
$NeuAc_1$ Fuc_1 Hex_7 $HexNAc_6$	3504	1.34	0.06	12.59*	*Product from 4226/4587
Fuc_1 Hex_8 $HexNAc_7$	3592	1.00	14.48	1.11	and α2,6-Sia
$NeuAc_2$ Fuc_1 Hex_6 $HexNAc_6$	3661	8.78	0.10	35.27*	*Product from 4022
$NeuAc_1$ Fuc_1 Hex_7 $HexNAc_7$	3749	0.57	0.08	6.83	and α2,6-Sia
Fuc_2 Hex_8 $HexNAc_7$	3766	1.18	0.50	0.64	
$NeuAc_3$ Fuc_1 Hex_6 $HexNAc_5$	3777	12.24	0.05	2.35	
Fuc_1 Hex_9 $HexNAc_7$	3796	0.67	1.03	0.18	
Fuc_1 Hex_8 $HexNAc_8$	3837	1.97	0.20	0.02	
$NeuAc_2$ Fuc_1 Hex_7 $HexNAc_6$	3865	6.43	0.09**	9.04	**α2,6-Sia

Supplementary Table 4

List of representative peaks of N-glycans by MALDI-TOF-MS analysis in SIAT1 cell line

Composition	Mass (m/z)	SIAT1<br>Non<br>%	 NeuA<br>%	 NeuS<br>%	Remarks
Hex_9 $HexNAc_8$	3867	6.67	1.06	2.90	
$NeuAc_1$ Fuc_1 Hex_8 $HexNAc_7$	3953	0.88	0.08	6.87	
$NeuAc_3$ Fuc_1 Hex_6 $HexNAc_6$	4022	18.94	0.09**	1.36**	**α2,3- and α2,6-Sia
Fuc_1 Hex_9 $HexNAc_8$	4041	0.73	7.00	0.33	
$NeuAc_2$ Fuc_1 Hex_7 $HexNAc_7$	4111	0.91	0.07	3.03	
Fuc_2 Hex_9 $HexNAc_8$	4215	0.11	0.31	0.11	
$NeuAc_3$ Fuc_1 Hex_7 $HexNAc_6$	4226	2.67	0.08	0.71	
Fuc_1 Hex_{10} $HexNAc_8$	4246	0.09	0.25	0.03	
Fuc_1 Hex_9 $HexNAc_9$	4286	0.19	0.23	0.06	
$NeuAc_2$ Fuc_1 Hex_8 $HexNAc_7$	4314	1.41	0.17	4.34	
$NeuAc_1$ Fuc_1 Hex_9 $HexNAc_8$	4403	0.50	0.07	0.47	
$NeuAc_3$ Fuc_1 Hex_7 $HexNAc_7$	4471	1.29	0.06	0.27	
Fuc_1 Hex_{10} $HexNAc_9$	4491	0.19	0.95	0.08	
$NeuAc_2$ Hex_8 $HexNAc_8$	4559	0.15	0.06	0.75	
$NeuAc_4$ Fuc_1 Hex_7 $HexNAc_6$	4587	1.98	0.07	0.20	
Fuc_2 Hex_{10} $HexNAc_9$	4665	0.14	0.11	0.06	
$NeuAc_3$ Fuc_1 Hex_8 $HexNAc_7$	4675	3.68	0.06	0.16	
Fuc_1 Hex_{11} $HexNAc_9$	4696	0.08	0.09	0.04	
$NeuAc_2$ Fuc_1 Hex_9 $HexNAc_8$	4764	0.12	0.08	2.36	
Fuc_1 Hex_{11} $HexNAc_{10}$	4939	0.05	0.19	0.03	
$NeuAc_4$ Fuc_1 Hex_8 $HexNAc_7$	5036	0.86	0.06	0.09	
$NeuAc_3$ Fuc_1 Hex_9 $HexNAc_8$	5125	1.22	0.06	1.06	
$NeuAc_2$ Fuc_1 Hex_{10} $HexNAc_9$	5213	0.04	0.07	0.17	
Fuc_1 Hex_{12} $HexNAc_{11}$	5388	0.02	0.08	0.03	
$NeuAc_4$ Fuc_1 Hex_9 $HexNAc_8$	5487	0.34	0.05	0.03	
$NeuAc_3$ Fuc_1 Hex_{10} $HexNAc_9$	5574	0.06	0.10	0.08	
Fuc_1 Hex_{13} $HexNAc_{12}$	5838	0.01	0.05	0.02	
$NeuAc_4$ Fuc_1 Hex_{10} $HexNAc_9$	5935	0.10	0.07	0.03	
