# Supplementary material for: Sialylated and sulfated N-Glycans in MDCK and engineered MDCK cells for influenza virus studies
Source: Sci Rep. 2022 Jul 26;12:12757. doi: 10.1038/s41598-022-16605-5 (PMC9325728; doi:10.1038/s41598-022-16605-5)
Supplement: Supplementary file 5 — Supplementary Table S5. [file 41598_2022_16605_MOESM5_ESM.pdf]

Supplementary Table 5

List of representative peaks of N-glycans by MALDI-TOF-MS analysis in hCK cell line

Composition	Mass (m/z)	hCK Non %	hCK NeuA %	hCK NeuS %	Remarks
$Hex_5\ HexNAc_2$	1580	43.64	42.30	25.07	
$Hex_6\ HexNAc_2$	1784	64.88	74.67	49.50	
$Fuc_1\ Hex_3\ HexNAc_4$	1836	12.58	6.81	3.32	
$Hex_7\ HexNAc_2$	1988	73.28	65.83	52.72	
$Hex_5\ HexNAc_4$	2070	5.31	45.39	5.01	
$Fuc_1\ Hex_3\ HexNAc_5$	2081	14.62	6.67	0.23	
$NeuAc_1\ Fuc_1\ Hex_4\ HexNAc_3$	2156	17.68	0.20	6.29	
$Hex_8\ HexNAc_2$	2192	88.59	78.37	67.43	
$Fuc_1\ Hex_5\ HexNAc_4$	2244	5.61	91.03*	6.87	*Product from 2605/2966
$Fuc_1\ Hex_4\ HexNAc_5$	2285	2.81	18.77	0.59	
$Fuc_1\ Hex_3\ HexNAc_6$	2326	9.37	6.00	1.93	
$Hex_9\ HexNAc_2$	2396	100.00	100.00	100.00	**Relative intensity**
$Fuc_1\ Hex_5\ HexNAc_5$	2489	1.21	37.21*	0.46	*Product from 2850/3212
$Hex_6\ HexNAc_5$	2519	0.75	9.58	0.21	
$NeuAc_1\ Fuc_1\ Hex_5\ HexNAc_4$	2605	9.61	0.37	3.01	
$NeuAc_1\ Fuc_1\ Hex_4\ HexNAc_5$	2646	8.25	0.03	3.48	
$Fuc_1\ Hex_6\ HexNAc_5$	2693	0.95	33.33*	0.39	*Product from 3054/3777
$Fuc_1\ Hex_5\ HexNAc_6$	2734	1.02	7.71	0.29	
$Hex_6\ HexNAc_6$	2764	0.64	14.32	0.18	
$NeuAc_2\ Hex_5\ HexNAc_4$	2792	6.87	0.04**	5.47	**α2,6-Sia
$NeuAc_1\ Fuc_1\ Hex_5\ HexNAc_5$	2850	4.52	0.03	1.60	
$Fuc_1\ Hex_6\ HexNAc_6$	2938	0.67	42.96	0.30	*Product from 3661/4022
$NeuAc_2\ Fuc_1\ Hex_5\ HexNAc_4$	2966	22.40	0.23**	29.23	**α2,6-Sia
$Hex_7\ HexNAc_6$	2968	12.75	31.10	19.93	
$NeuAc_1\ Fuc_1\ Hex_6\ HexNAc_5$	3054	2.78	0.05	1.98	
$Fuc_1\ Hex_7\ HexNAc_6$	3143	0.79	82.43*	0.39	*Product from 3504/3865
$NeuAc_2\ Fuc_1\ Hex_5\ HexNAc_5$	3212	11.33	0.08**	7.81	**α2,6-Sia
$NeuAc_1\ Fuc_1\ Hex_6\ HexNAc_6$	3298	2.69	0.08	1.08	
$Fuc_2\ Hex_7\ HexNAc_6$	3317	0.87	2.36	0.20	
$Fuc_1\ Hex_8\ HexNAc_6$	3347	0.29	17.16	0.09	
$Fuc_1\ Hex_7\ HexNAc_7$	3388	0.27	19.15	0.12	
$NeuAc_2\ Fuc_1\ Hex_6\ HexNAc_5$	3416	2.86	0.08**	4.42	**α2,6-Sia
$Hex_8\ HexNAc_7$	3418	2.86	3.72	2.33	
$NeuAc_1\ Fuc_1\ Hex_7\ HexNAc_6$	3504	2.67	0.03**	2.99	**α2,6-Sia
$Fuc_1\ Hex_8\ HexNAc_7$	3592	0.43	26.88	0.48	
$NeuAc_2\ Fuc_1\ Hex_6\ HexNAc_6$	3661	3.51	0.17**	4.32	**α2,6-Sia
$Fuc_2\ Hex_8\ HexNAc_7$	3766	1.83	1.68	0.08	
$NeuAc_3\ Fuc_1\ Hex_6\ HexNAc_5$	3777	2.79	0.02	1.86	
$Fuc_1\ Hex_9\ HexNAc_7$	3796	0.35	4.35	0.08	
$Hex_9\ HexNAc_8$	3837	0.37	0.72	0.29	
$NeuAc_2\ Fuc_1\ Hex_7\ HexNAc_6$	3865	6.71	0.02**	7.81	**α2,6-Sia
$Hex_9\ HexNAc_8$	3867	9.75	2.55	2.34	
$NeuAc_3\ Fuc_1\ Hex_6\ HexNAc_6$	4022	1.82	0.03	0.67	**α2,3- and α2,6-Sia

Supplementary Table 5

List of representative peaks of N-glycans by MALDI-TOF-MS analysis in hCK cell line

Composition	Mass (m/z)	hCK Non %	hCK NeuA %	hCK NeuS %	Remarks
$Fuc_1\ Hex_9\ HexNAc_8$	4041	1.07	13.24	0.33	
$NeuAc_2\ Fuc_1\ Hex_8\ HexNAc_6$	4069	1.15	0.10	1.15	
$Fuc_2\ Hex_9\ HexNAc_8$	4215	0.36	0.77	0.03	
$NeuAc_3\ Fuc_1\ Hex_7\ HexNAc_6$	4226	3.97	0.08	2.45	
$Fuc_1\ Hex_{10}\ HexNAc_8$	4246	0.24	0.73	0.04	
$Fuc_1\ Hex_9\ HexNAc_9$	4286	0.12	0.51	0.01	
$NeuAc_2\ Fuc_1\ Hex_8\ HexNAc_7$	4314	1.77	0.04	0.86	
$Hex_{10}\ HexNAc_9$	4316	0.40	1.21	0.74	**α2,3- and α2,6-Sia
$Fuc_1\ Hex_{10}\ HexNAc_9$	4491	0.19	5.62	0.07	
$NeuAc_4\ Fuc_1\ Hex_7\ HexNAc_6$	4587	1.64	0.02**	0.05**	**α2,3- and α2,6-Sia
$Fuc_2\ Hex_{10}\ HexNAc_9$	4665	0.02	0.24	0.03	
$NeuAc_3\ Fuc_1\ Hex_8\ HexNAc_7$	4675	2.00	0.03	0.66	
$Fuc_1\ Hex_{10}\ HexNAc_9$	4695	0.01	0.12	0.01	
$NeuAc_2\ Fuc_1\ Hex_9\ HexNAc_8$	4763	0.08	0.18	0.15	
$Fuc_1\ Hex_{11}\ HexNAc_{10}$	4939	0.02	1.89	0.02	
$NeuAc_3\ Fuc_1\ Hex_9\ HexNAc_8$	5125	0.46	0.03	0.22	
$Fuc_1\ Hex_{12}\ HexNAc_{11}$	5388	0.01	0.16	0.02	
$NeuAc_3\ Fuc_1\ Hex_{10}\ HexNAc_9$	5574	0.03	0.02	0.05	
$Fuc_1\ Hex_{13}\ HexNAc_{12}$	5838	< 0.01	0.02	0.02	
