# Supplementary material for: Sialylated and sulfated N-Glycans in MDCK and engineered MDCK cells for influenza virus studies
Source: Sci Rep. 2022 Jul 26;12:12757. doi: 10.1038/s41598-022-16605-5 (PMC9325728; doi:10.1038/s41598-022-16605-5)
Supplement: Supplementary file 6 — Supplementary Legends. [file 41598_2022_16605_MOESM6_ESM.docx]

**Byrd-Leotis, Jia et al.**

**Supplementary Information**

**Supplementary Table Legends**

**Supplementary Table S1–** List of assigned peaks of N-glycans by MALDI-TOF-MS analysis in cell lines.

**Supplementary Table S2-** Percentage values of structural features (%).

**Supplementary Table S3-** List of representative peaks of N-glycans by MALDI-TOF-MS analysis in MDCK cell line.

**Supplementary Table S4-** List of representative peaks of N-glycans by MALDI-TOF-MS analysis in SIAT1 cell line.

**Supplementary Table S5-** List of representative peaks of N-glycans by MALDI-TOF-MS analysis in hCK cell line.

**Supplementary** **Figure Legends**

**Supplementary Figure S1-** Original, uncut blots and gels used in main Figure 4 for passage 3.

**Supplementary Figure S2-** Original, uncut blots and gels used in main Figure 4 for passage 23.
