# Supplementary figures and images for: Sialylated and sulfated N-Glycans in MDCK and engineered MDCK cells for influenza virus studies
Source: Sci Rep. 2022 Jul 26;12:12757. doi: 10.1038/s41598-022-16605-5 (PMC9325728; doi:10.1038/s41598-022-16605-5)

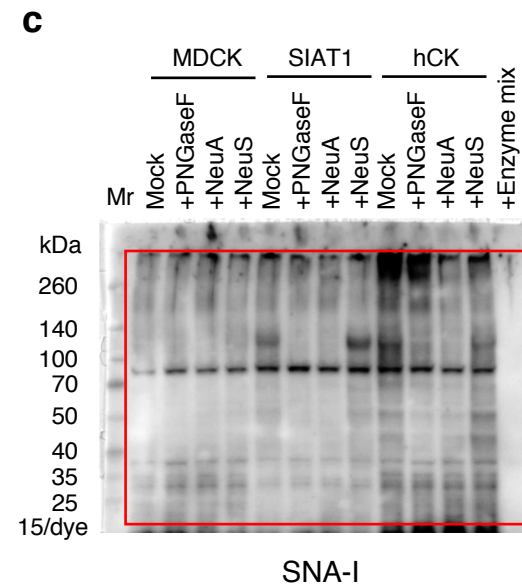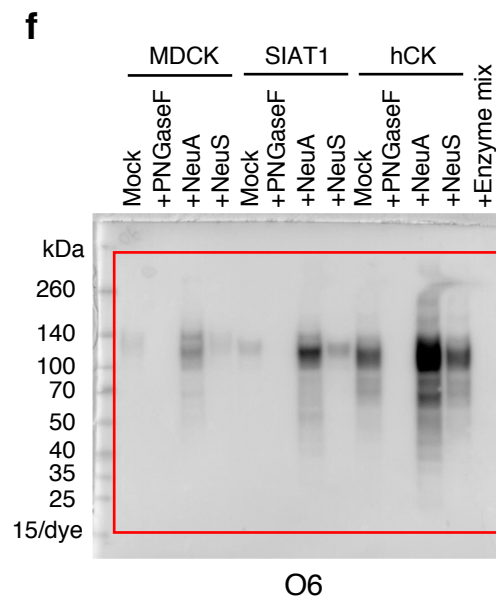

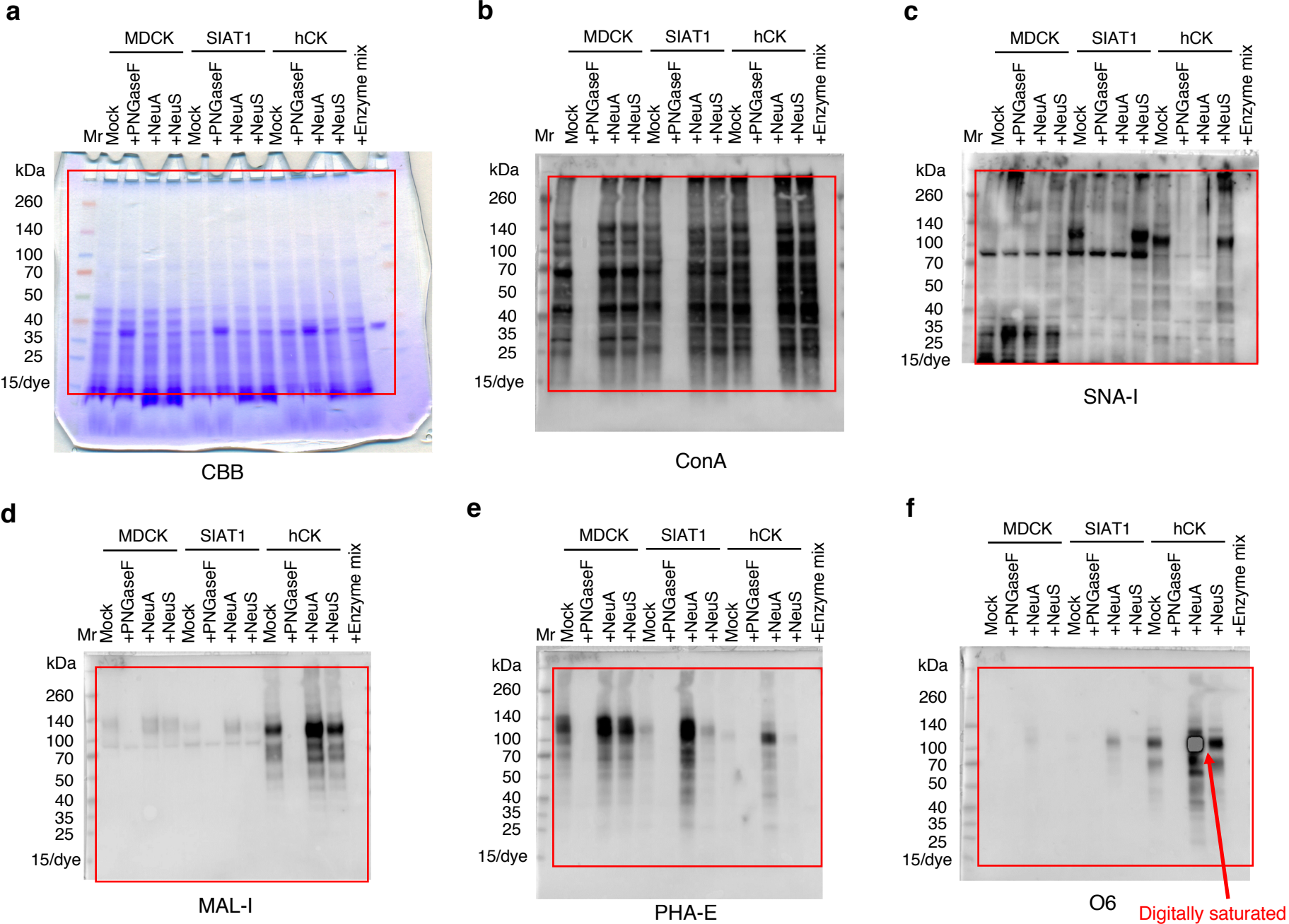

Supplement: Supplementary file 7 — Supplementary Figures. [file 41598_2022_16605_MOESM7_ESM.pdf]
